# Dynamic role of CUL4B in radiation-induced intestinal injury-regeneration

**DOI:** 10.1038/s41598-024-60704-4

**Published:** 2024-04-30

**Authors:** Beibei Guo, Xiaohan Huo, Xueyong Xie, Xiaohui Zhang, Jiabei Lian, Xiyu Zhang, Yaoqin Gong, Hao Dou, Yujia Fan, Yunuo Mao, Jinshen Wang, Huili Hu

**Affiliations:** 1 The Key Laboratory of Experimental Teratology, Ministry of Education and Department of Systems Biomedicine and Research, School of Basic Medical Sciences, Cheeloo Medical College, Shandong University, Jinan, 250012 China; 2 The Key Laboratory of Experimental Teratology, Ministry of Education and Department of Molecular Medicine and Genetics, School of Basic Medical Sciences, Shandong University Cheeloo Medical College, Jinan, 250012 China; 3 Department of Gastrointestinal Surgery, Shandong Provincial Hospital Affiliated to Shandong First Medical University, Jinan, 250021 Shandong China

**Keywords:** CUL4B, Ionizing radiation, Intestinal injury-regeneration, p53-mediated apoptosis, Gastroenterology, Molecular biology

## Abstract

CUL4B, a crucial scaffolding protein in the largest E3 ubiquitin ligase complex CRL4B, is involved in a broad range of physiological and pathological processes. While previous research has shown that CUL4B participates in maintaining intestinal homeostasis and function, its involvement in facilitating intestinal recovery following ionizing radiation (IR) damage has not been fully elucidated. Here, we utilized in vivo and in vitro models to decipher the role of CUL4B in intestinal repair after IR-injury. Our findings demonstrated that prior to radiation exposure, CUL4B inhibited the ubiquitination modification of PSME3, which led to the accumulation of PSME3 and subsequent negative regulation of p53-mediated apoptosis. In contrast, after radiation, CUL4B dissociated from PSME3 and translocated into the nucleus at phosphorylated histones H2A (γH2AX) foci, thereby impeding DNA damage repair and augmenting p53-mediated apoptosis through inhibition of BRCA1 phosphorylation and RAD51. Our study elucidated the dynamic role of CUL4B in the repair of radiation-induced intestinal damage and uncovered novel molecular mechanisms underlying the repair process, suggesting a potential therapeutic strategy of intestinal damage after radiation therapy for cancers.

## Introduction

The toxicity of ionizing radiation (IR) is usually associated with chronic and acute radiation syndromes that occur following exposure to IR, resulting in damage to a wide range of organs such as the stomach and intestine. In case of intestine, active Lgr5^+^ intestinal stem cells (ISCs) are responsible for governing the self-renewal of epithelial cells under normal conditions. But they are simultaneously affected by IR-induced ablation, leading to loss of crypts and disruption of crypt-villus structures^1^. Numerous researches have attempted to elucidate the molecular mechanisms involved in the repair of intestinal damage after IR exposure. It has been shown that IR primarily leads to DNA double-strand breaks (DSBs) and determines the fate of cells after IR inducted by discrepancy of intracellular molecular levels, wherein p53 identified as a key protein in the cellular damage process induced by IR^2^. The activation and accumulation of p53 regulate a wide range of responsive genes, including PUMA-NOXA-BAX^3–5^ and p21^6^, resulting in cell apoptosis and irreversible cell cycle arrest, respectively. Moreover, γH2AX (serine 139-phosporylated state) is commonly recognized as a biomarker for DNA damage^7^ and is responsible to recruit other DNA repair proteins, such as BRCA1^8^, p53-binding protein 1 (53BP1)^9^, mediator of DNA damage checkpoint protein 1 (MDC1)^10^ and ataxia-telangiectasia mutated (ATM)^11^. Intriguingly, recent studies indicate that γH2AX is also a decision-maker in apoptosis and survival^12,13^.

CUL4B belongs to the Cullin family which are scaffolding proteins for the E3 ubiquitin ligase complex CRLs, acting as a pivotal role in cell cycle regulation, DNA replication and DNA damage repair (DDR)^14^. Under physiological conditions, CUL4B regulates intestinal homeostasis and ISCs self-renewal by targeting immune-related GTPase family M member 1 (IRGM1) for proteasomal degradation^15^. Under UV-pathological conditions, DDB1-CUL4B^DDB2^, an individual E3 ligase, possesses higher efficiency in monoubiquitinating histone H2A after recognizing UV-damaged DNA lesions^16,17^. In addition, CUL4B acts as an essential E3 ligase of CRL4B^RBBP7^ responsible for proteasomal degradation by ubiquitinating HUWE1 in a NEDD8-dependent manner^18,19^. However, the precise function of CUL4B in radiation-induced intestinal injury and its underlying molecular mechanism are not fully understood.

In this study, we aimed to elucidate the dynamic function of CUL4B in the context of intestinal damage induced by IR. Before IR exposure, the deletion of CUL4B led to the downregulation of its binding protein PSME3, which enhanced p53 and subsequent promotion of apoptosis. After IR exposure, CUL4B translocated to the nucleus and localized at the γH2AX foci, leading to elevated p53 expression and inhibition of the damage recovery process. Our findings successfully explored the specific target molecules and mechanisms through which CUL4B is involved in the intestinal repair following IR treatment, offering potential insights for modulating intestinal damage repair process.

## Results

### Depletion of CUL4B increases intestinal sensitivity to IR-induced damage

**Figure 1:**
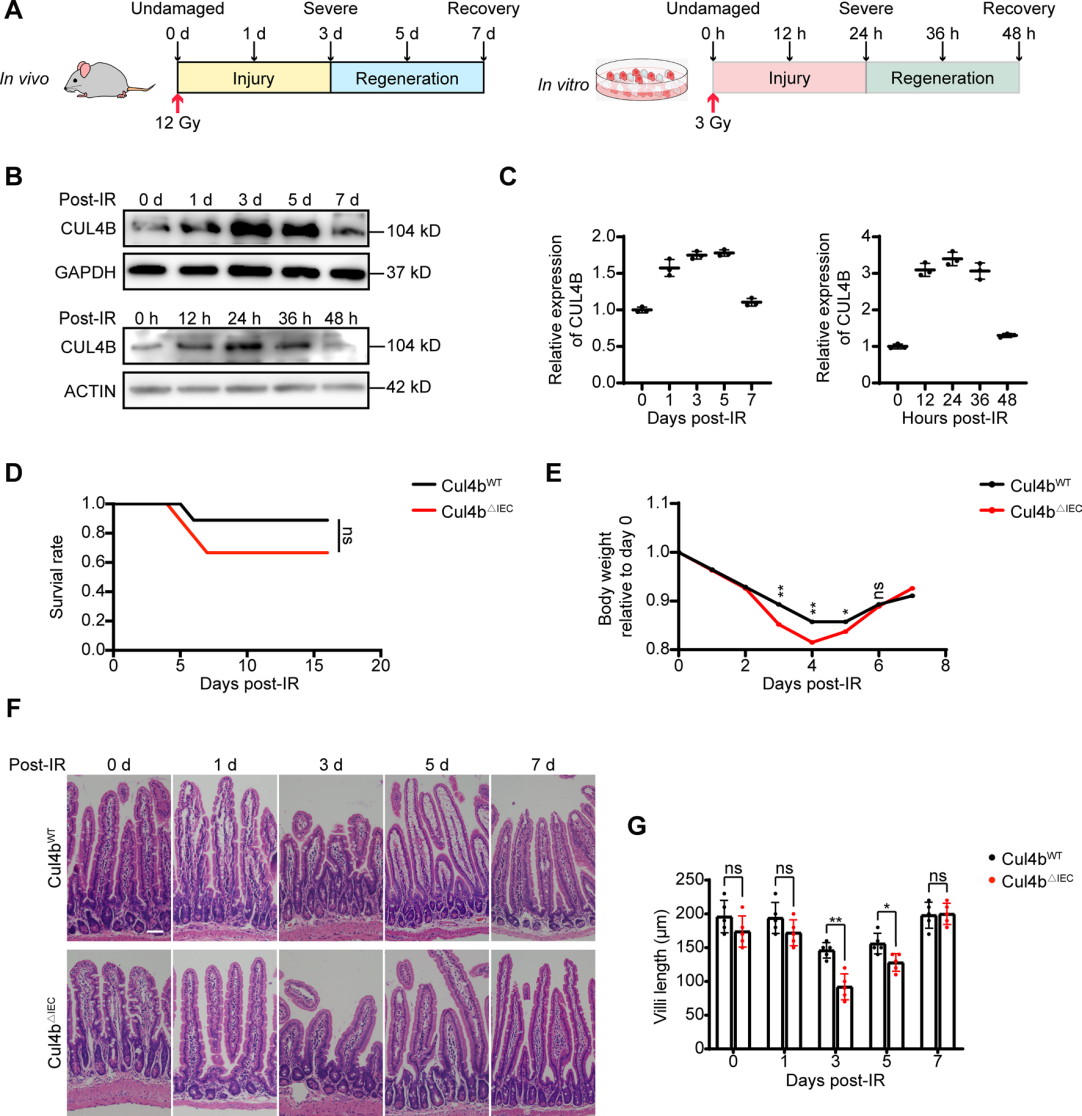
CUL4B disrupted IR induced intestinal damage and recovery. (**A**) Schematic overview of the mice (*Cul4b*^*WT*^ and *Cul4b*^*ΔIEC*^) model and the IEC-6 cells (sh*Cul4b* and shNC). Mice were sacrificed at 0, 1, 3, 5 and 7 days after 12 Gy whole-body irradiation and cells were collected at 0, 12, 24, 36 and 48 h after 3 Gy irradiation. (**B**, **C**) Western blot analysis of CUL4B expression in intestine of *Cul4b*^*WT*^ mice (n = 3) after radiation at 0, 1, 3, 5 and 7 days and in IEC-6 cells after radiation at 0, 12, 24, 36 and 48 h (**B**) and statistical analysis (**C**). Three independent experiments were performed. Error bars represent SD. (**D**) Overall survival analysis of the *Cul4b*^*WT*^ and *Cul4b*^*ΔIEC*^ mice post-irradiation. *ns* not significant, based on Log-rank test. (**E)** Statistical analysis of the body weight of the *Cul4b*^*WT*^ and *Cul4b*^*ΔIEC*^ mice post-irradiation relative to day 0. (**F**, **G**) Representative H&E images of *Cul4b*^*WT*^ and *Cul4b*^*ΔIEC*^ mice after radiation and the quantification of the length of crypt-villi (3 vs. 3) (**F**) and statistical analysis of villi length (**G**). Scale Bar = 50 μm. Error bars represent SD, *P < 0.05; **P < 0.01; *ns* not significant, based on Student’s *t* test. The original western blots are presented in Fig. S6.

In order to explore the function of CUL4B in intestinal recovery from IR-induced damage, we successfully constructed models by exposing mice and IEC-6 cells to non-lethal doses of irradiation, respectively (Fig. 1A) (see “Methods” section). Our analysis revealed a significant increase in CUL4B expression following irradiation, which subsequently returned to baseline levels at 7 days post-irradiation in mice. Similarly, CUL4B expression peaked at 24 h post-irradiation in IEC-6 cells, and recovered to the unirradiated level at 48 h, implying that CUL4B plays a critical role in intestinal recovery process (Fig. 1B, C).

To further explore the function of CUL4B in intestinal recovery, we constructed epithelium-specific CUL4B deletion mice (*Cul4b*^*ΔIEC*^), and the knockout efficiency was confirmed at both mRNA and protein levels (Fig. S1A-S1B). Although no significant survival rate following IR treatment was exhibited in *Cul4b*^*ΔIEC*^ mice, they showed a higher degree of impairment in terms of body weight loss and villi/crypt length abnormalities compared to control mice (*Cul4b*^*WT*^) 3-5 days after IR (Fig. 1D-G). 7 days after radiation, the weight and intestinal length recovered approximately comparable to the control (Fig. 1E-G). These findings suggest that the deletion of CUL4B enhances the radiosensitivity of the intestine.

### CUL4B deficiency facilitates intestinal recovery from IR-induced damage via suppression of cell apoptosis

As mentioned above, the absence of CUL4B resulted in more severe intestinal damage after radiation exposure, but the intestine possessed greater resilience compared to *Cul4b*^*WT*^ mice. To investigate the function of CUL4B in intestinal damage repair, we first examined the proliferative signals in regenerating intestines after IR injury.

**Figure 2:**
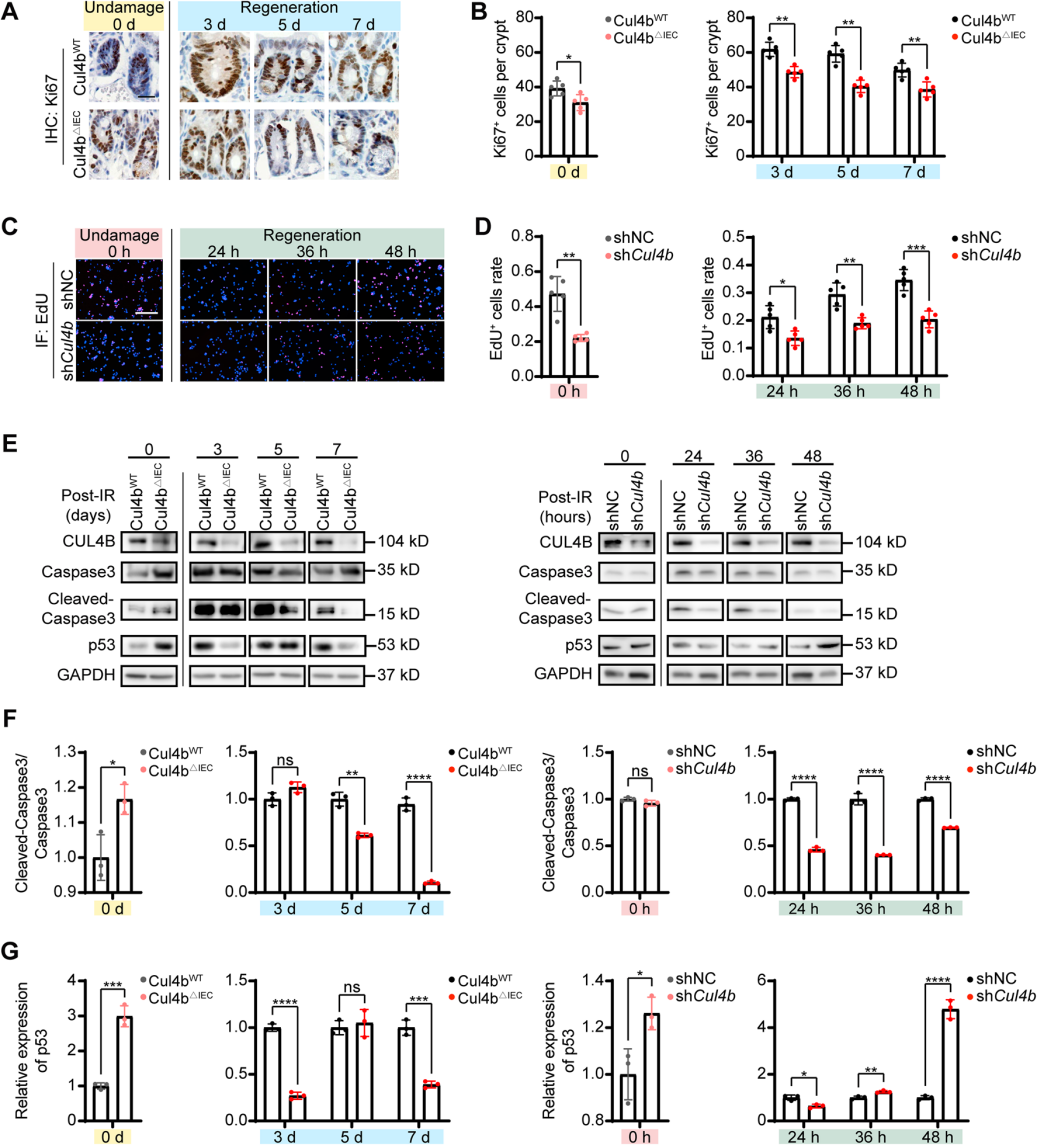
CUL4B deficiency downregulated cell apoptosis post-IR. (**A**, **B**) Representative images and statistical analysis of immunochemical staining of Ki67^+^ cells per crypt in intestine from *Cul4b*^*WT*^ and *Cul4b*^*ΔIEC*^ mice (**A**) and statistical analysis (**B**). Scale Bar = 20 μm. Error bars represent SD, *P < 0.05; **P < 0.01; *ns* not significant, based on Student’s *t* test. (**C**, **D**) Representative images and statistical analysis of immunofluorescent staining of EdU^+^ cells in sh*Cul4b* and shNC IEC-6 cells (**C**) and statistical analysis (**D**). Blue: DAPI; Red: EdU^+^. Scale Bar = 200 μm. Three independent experiments were performed. Error bars represent SD, *P < 0.05; **P < 0.01; ***P < 0.001, based on Student’s *t* test. (**E**-**G**) Western blot and statistical analysis of Caspase3, Cleaved-Caspase3 and p53 expression relative to GAPDH in intestine from *Cul4b*^*WT*^ and *Cul4b*^*ΔIEC*^ mice after radiation at 0, 1, 3, 5 and 7 days and in sh*Cul4b* and shNC IEC-6 cells after radiation at 0, 12, 24, 36 and 48 h. Three independent experiments were performed. Error bars represent SD, *P < 0.05; **P < 0.01; ***P < 0.001; ****P < 0.0001; *ns* not significant, based on Student’s *t* test. The original western blots are presented in Fig. S6.

We analyzed the number of Ki67^+^ proliferative cells and found that Ki67 expression was significantly elevated after intestinal injury induced repair in *Cul4b*^*WT*^ mice, while there was no higher expression of Ki67 in *Cul4b*^*ΔIEC*^ mice (Fig. 2A, B). Similarly, we did not detect more pronounced proliferation by EdU staining of sh*Cul4b* IEC-6 cells during regeneration (Fig. 2C, D). As shown in Fig. S2, the expression of inflammation factors such as *Tnfα*, *Il6* and *Il1β* displayed unchanged or similar expression profiles in *Cul4b* deficiency mice before and after IR. Moreover, the expression of key proteins in cGAS/STING signaling, which bridges the IR-induced DNA damage with immune activation, was not changed. Subsequently, we further examined the alterations in apoptosis during intestinal recovery. Notably, CUL4B deletion led to an increase in Cleaved Caspase3/Caspase3 in intestinal cells in mice without radiation. Conversely, CUL4B deletion resulted in an attenuated apoptosis in mice during intestinal recovery, as evidenced by a decrease of Cleaved Caspase3/Caspase3 (Fig. 2E, F). The contrasting pattern of apoptosis under CUL4B deletion pre- and post-radiation was also verified in cell lines (Fig. 2E, F, Fig. S3). Above results suggest that CUL4B is involved in the intestinal recovery from radiation-induced damage by orchestrating cell apoptosis process.

### CUL4B manipulates intestinal repair of injury through p53-mediated apoptosis

Given that the intestinal repair after IR injury proceeds via a p53-dependent pathway^20^, we evaluated the p53 expression in both CUL4B-deficient mice and cell lines to further investigate the involvement of CUL4B in the regulation of apoptosis through the p53 signaling pathway in intestinal injury repair. Surprisingly, CUL4B deficiency dramatically elevated the p53 protein expression in non-irradiated mice, but downregulated p53 protein levels during intestinal recovery process (Fig. 2E, G). Thus, we speculated that CUL4B could attenuate p53-mediated apoptosis in the absence of IR injury, whereas promote p53-mediated apoptosis during repair of intestinal injury.

**Figure 3:**
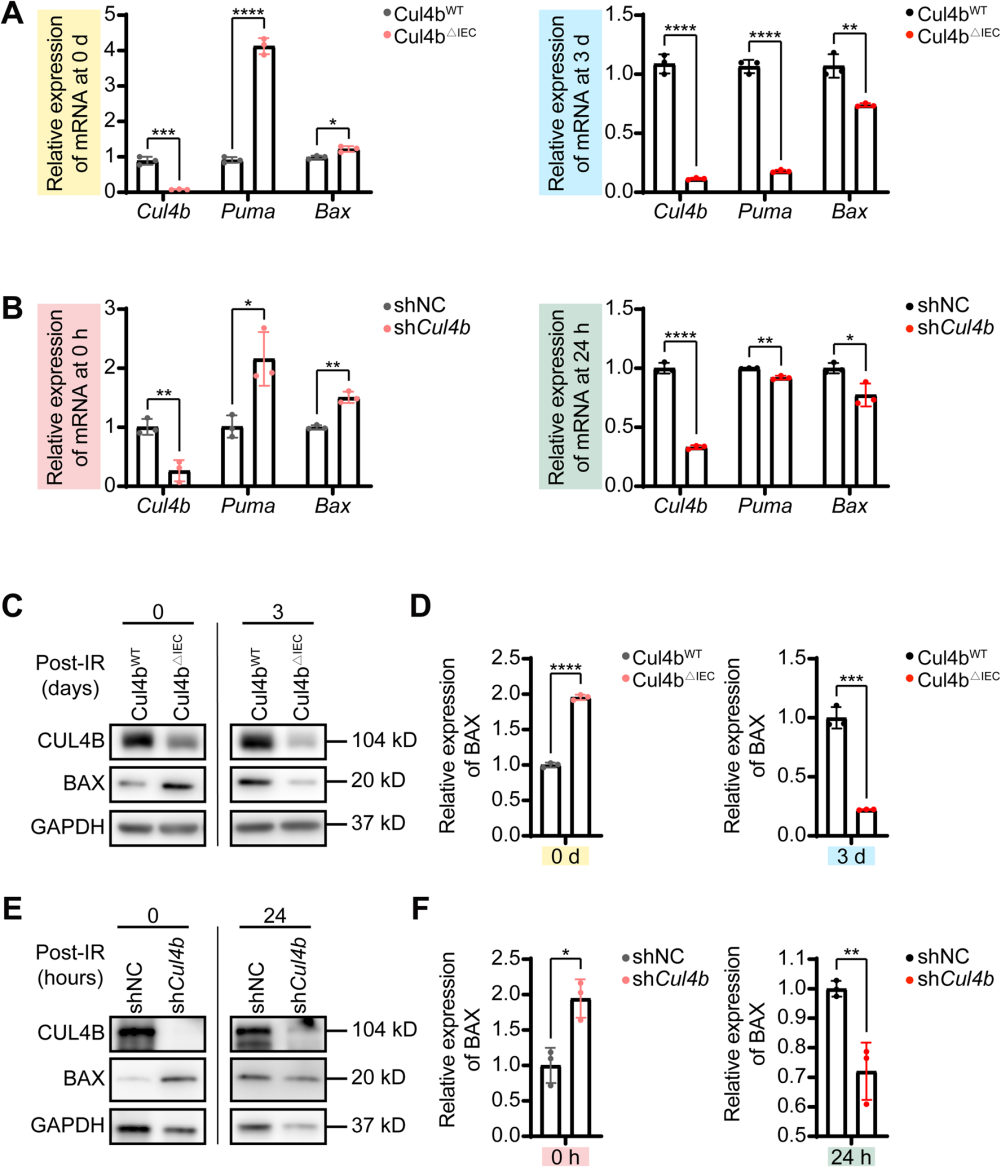
Apoptosis associated p53 downstream genes were regulated by CUL4B. (**A**) RT-qPCR analysis of  *Puma* and *Bax* expression in *Cul4b*^*WT*^ and *Cul4b*^*ΔIEC*^ mice before and after radiation at 3 days. Error bars represent SD, *P < 0.05; **P < 0.01; ***P < 0.001; ****P < 0.0001, based on Student’s *t* test. (**B**) RT-qPCR analysis of *Puma* and *Bax* expression in sh*Cul4b* and shNC IEC-6 cells before and after radiation at 24 h. Error bars represent SD, *P < 0.05; **P < 0.01; ****P < 0.0001, based on Student’s *t* test. (**C**, **D**) Western blot and statistical analysis of CUL4B and BAX expression in intestine from *Cul4b*^*WT*^ and *Cul4b*^*ΔIEC*^ mice before and after radiation at 3 days. Error bars represent SD, ***P < 0.001; ****P < 0.0001, based on Student’s *t* test. (**E**, **F**) Western blot and statistical analysis of CUL4B and BAX expression in sh*Cul4b* and shNC IEC-6 cells before and after radiation at 24 h. Error bars represent SD, *P < 0.05; **P < 0.01, based on Student’s *t* test. The original western blots are presented in Fig. S6.

To further verify this hypothesis, we detected the downstream target genes of p53, *Puma* (p53 upregulated modulator of apoptosis) and *Bax* (BCL2 associated X, apoptosis regulator), through RT-qPCR and Western blot. Accordingly, RT-qPCR results showed that, consistent with the pattern of p53 expression, *Puma* and *Bax* were significantly upregulated in CUL4B-deficient, non-IR induced mice and IEC-6 cells, but expressed less in CUL4B-deficient, IR induced injury models (Fig. 3A, B). Similar changes were observed at the protein level (Fig. 3C-F). In brief, the coordinated expression of CUL4B and p53 protein, together with the apparent variation in p53 downstream gene expression, demonstrate that CUL4B could orchestrate intestinal injury repair through p53-mediated apoptosis.

### PSME3 synergizes with CUL4B before IR to modulate p53-mediated apoptosis

**Figure 4:**
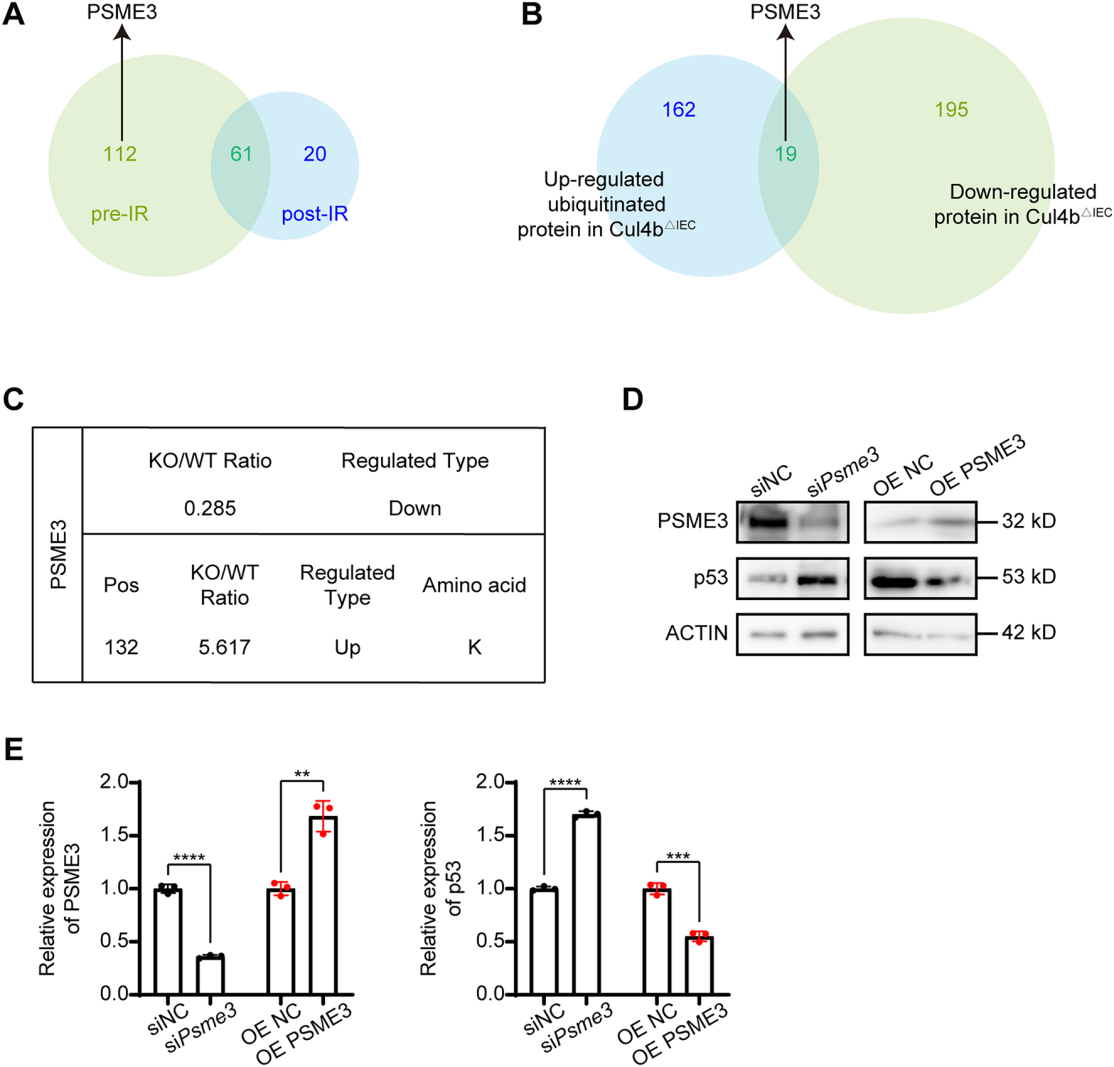
PSME3 bridged CUL4B and its regulation of p53. (**A**) Venn diagram of CUL4B binding-protein using MS in intestine of *Cul4b*^*WT*^ and *Cul4b*^*ΔIEC*^ mice pre-IR and post-IR at 3 days. (**B**) Venn diagram of the overlap of up-regulated ubiquitylated proteins and down-regulated proteins identified in *Cul4b*^*ΔIEC*^ mice compared with *Cul4b*^*WT*^ mice. (**C**) Changing folds and detail modification site of PSME3. (**D**, **E**) Western blot and statistical analysis of the expression of p53 in *Psme3*-knockdown (si*Psme3*) and PSME3-overexpressed (OE PSME3) IEC-6 cells relative to control (siNC and OE NC). Error bars represent SD, **P < 0.01; ***P < 0.001; ****P < 0.0001, based on Student’s *t* test. The original western blots are presented in Fig. S6.

To identify the key factor that incorporate CUL4B dynamically regulating p53-mediated apoptosis, mass spectrometry was conducted to analyze the proteins that interacted with CUL4B in both unirradiated and 3-day post-irradiation small intestine tissues of mice. A total of 173 CUL4B-binding proteins were detected in the absence of radiation, while 81 CUL4B-binding proteins were detected in 3-day post-irradiation samples (full lists in Table S1-S3). Among these, 61 proteins were found to consistently bind to CUL4B after irradiation, while 112 proteins initially bound to CUL4B, but dissociated after irradiation (Fig. 4A). We also comprehensively analyzed differentiated expressional proteins and ubiquitylated proteins in intestines with CUL4B deletion (MS data in PRIDE: PXD-021528). Notably, the PSME3 protein was uniquely observed as the candidate key regulator among these proteins (Fig. 4A, B). PSME3 is a member of proteasome activator complex that mediates protein degradation and essential for protein homeostasis^21^. It also indicated a decrease in PSME3 protein levels (0.285-fold) and an increase in its ubiquitination form (5.617-fold) in *Cul4b*^*ΔIEC*^ mice^15^ (Fig. 4C, full lists in Table S4-S6). As PSME3 can enhance MDM2-mediated ubiquitination and degradation of p53^22^, the protein level of p53 was measured in IEC-6 cells with *Psme3* knockdown and overexpression, respectively. Western blot results revealed an increase in p53 levels with *Psme3* knockdown, and a decrease with *Psme3* overexpression (Fig. 4D, E). The negative correlation between PSME3 and p53 protein levels, giving rise to the speculation that PSME3 may play a critical role in CUL4B-regulated cell apoptosis.

**Figure 5:**
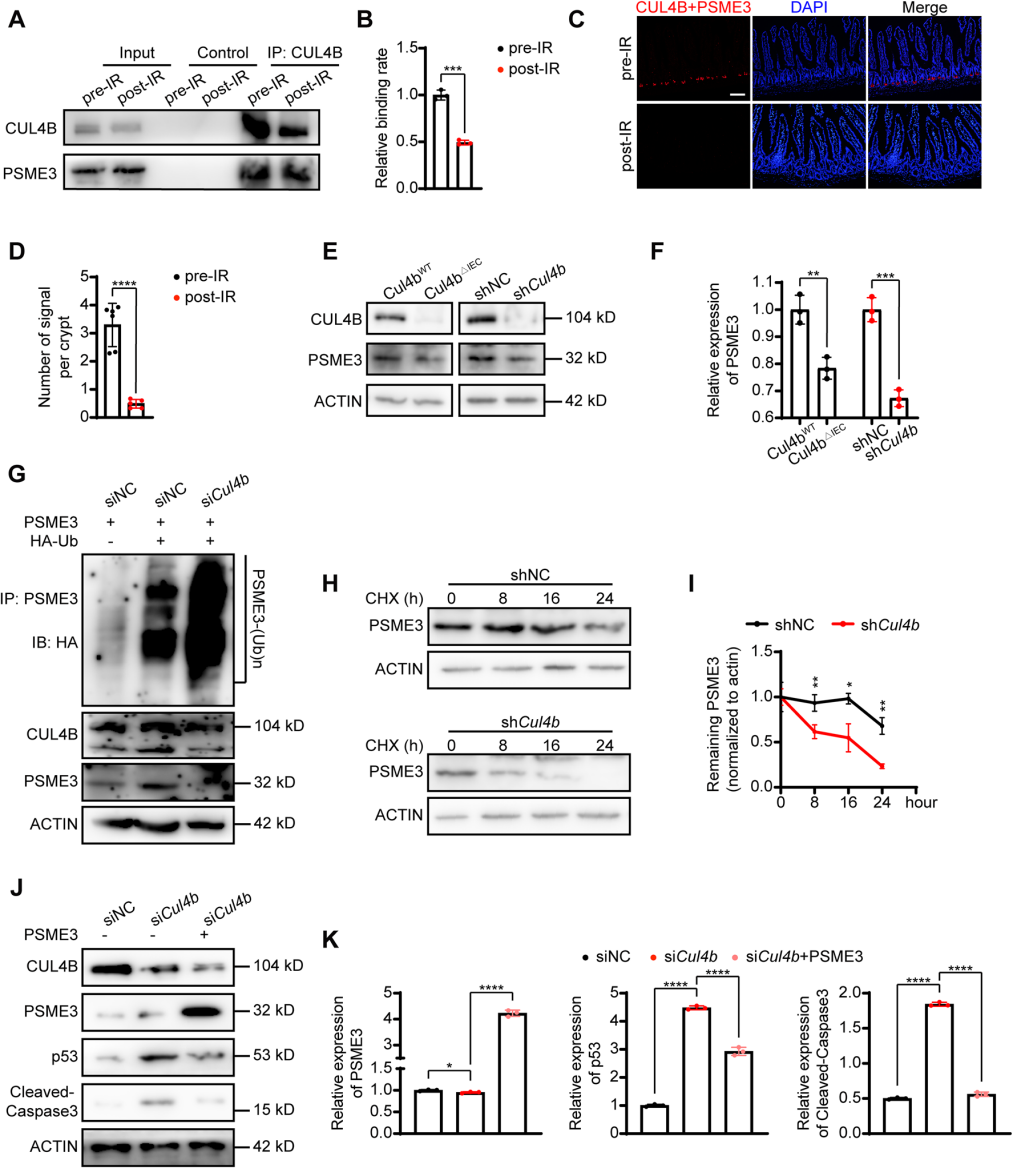
CUL4B deletion diminished PSME3 expression to up-regulate p53 before IR. (**A**, **B**) Immunoprecipitation and statistical analysis of the binding of CUL4B and PSME3 in intestines from *Cul4b*^*WT*^ mice pre-IR and post-IR at 3 days. Error bars represent SD, ***P < 0.001, based on Student’s *t* test. (**C**, **D**) Representative images and statistical analysis of PLA in intestine from *Cul4b*^*WT*^ mice pre-IR and post-IR at 3 days. Blue: DAPI; Red: binding signal. Scale Bar = 50 μm. Error bars represent SD, ****P < 0.0001, based on Student’s *t* test. (**E**, **F**) Western blot analysis and statistical analysis of PSME3 expression in *Cul4b*^*WT*^ and *Cul4b*^*ΔIEC*^ mice and sh*Cul4b* and shNC IEC-6 cells. Error bars represent SD, **P < 0.01; ***P < 0.001, based on Student’s *t* test. (**G**) Western blot analysis of the ubiquitination level of CUL4B on PSME3 in siNC and si*Cul4b* IEC-6 cells. (**H**, **I**) Western blot analysis and statistical analysis of the stability of PSME3 in sh*Cul4b* and shNC IEC-6 cells at 0, 8, 16 and 24 h after CHX treatment. Error bars represent SD, *P < 0.05; **P < 0.01, based on Student’s *t* test. (**J**, **K**) Western blot analysis of the P53, PSME3 and Cleaved-Caspase3 expression in siNC, si*Cul4b* and si*Cul4b* IEC-6 cells transfected with PSME3-expression plasmid. Error bars represent SD, *P < 0.05; ****P < 0.0001, based on Student’s *t* test. The original western blots are presented in Fig. S6.

To further investigate the role of PSME3 in CUL4B-coordinated repair of IR-damage intestine, we initially evaluated the binding of CUL4B to PSME3 pre- and post-irradiation. Immunoprecipitation experiments on intestinal tissues of both unirradiated and irradiated mice revealed a reduction in the amount of PSME3 combined with CUL4B after radiation (Fig. 5A, B). Coordinately, proximity ligation assay (PLA) was conducted on tissue slices of unirradiated and irradiated mice small intestine to visualize the interaction between CUL4B and PSME3. Consistently, the fluorescent signal that indicating the binding of CUL4B and PSME3 protein were significantly decreased after IR exposure (Fig. 5C, D). Moreover, PSME3 expression was downregulated in CUL4B deletion mice and IEC-6 cells, confirming the interaction between CUL4B and PSME3 (Fig. 5E, F).

Given the important role of CUL4B in ubiquitination modifications, the ubiquitination modification of PSME3 was further examined. It was found that the level of ubiquitination of PSME3 was increased in sh*Cul4b* IEC-6 cells, resulting in enhanced protein instability and shortened half-life (Fig. 5G-I), suggesting that CUL4B could inhibit PSME3 ubiquitination and thus maintain the long-term presence of PSME3. As mentioned above, overexpression of *Psme3* lead to the decrease of p53 expression. Therefore, we assume that CUL4B potentially regulating downstream p53 pathway activation through ubiquitination of PSME3. To verify this hypothesis, we detected whether the presence of PSME3 interfered with the effect of CUL4B on p53 expression. Addition of PSME3 in CUL4B deficiency cells significantly blocked the accumulation of p53 induced by CUL4B deletion (Fig. 5J, K), verifying that the presence of PSME3 inhibited the facilitation of p53 expression induced by CUL4B.

Taken together, PSME3 was a pivotal factor in the CUL4B modulation of p53-mediated apoptosis before IR, with CUL4B maintaining the existence of PSME3 by inhibiting its ubiquitination, and the persistent presence of PSME3 inhibiting the expression of p53, thereby suppressing the occurrence of apoptosis.

### CUL4B dissociated from PSME3 and bound to chromatin after IR damage discouraging the repair process

**Figure 6:**
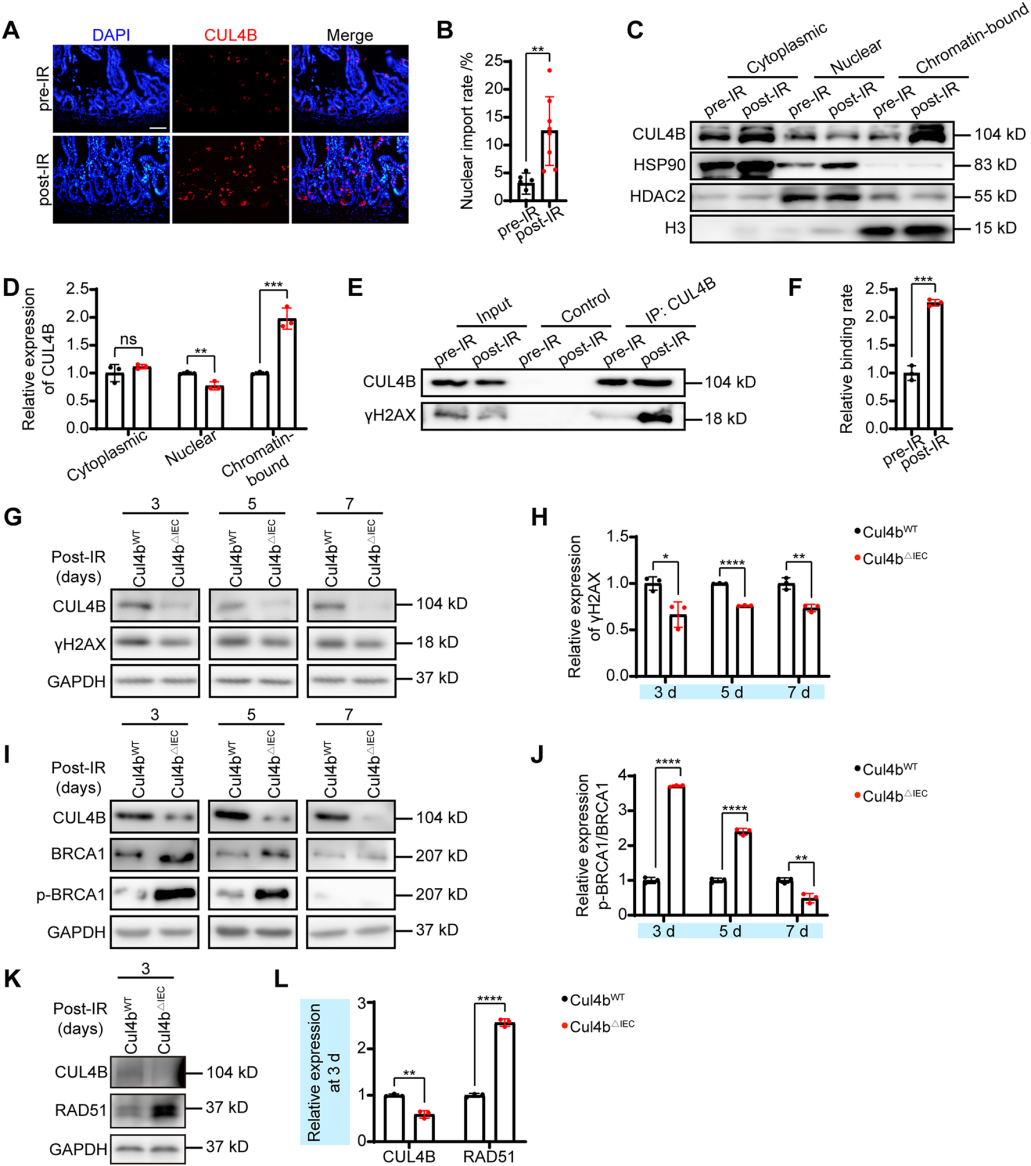
CUL4B converted subcellular localization and repressed DDR. (**A**, **B**) Representative images of immunofluorescent staining of CUL4B^+^ cells and statistical analysis of the rate of nuclear import of CUL4B pre-IR and post-IR at 3 days in intestine from *Cul4b*^*WT*^ mice. Scale Bar = 50 μm. Error bars represent SD, **P < 0.01, based on Student’s *t* test. (**C**, **D**) Western blot and statistical analysis of the CUL4B expression of cytoplasmic (HSP90 as control), nuclear (HDAC2 as control) and chromatin-bound (H3 as control) in intestine from *Cul4b*^*WT*^ mice pre-IR and post-IR at 3 days. Error bars represent SD, **P < 0.01; ***P < 0.001; *ns* not significant, based on Student’s *t* test. (**E**, **F**) Immunoprecipitation analysis of CUL4B and γH2AX in intestine from *Cul4b*^*WT*^ mice pre-IR and post-IR at 3 days. Error bars represent SD, ***P < 0.001, based on Student’s *t* test. (**G**, **H**) Western blot and statistical analysis of CUL4B and γH2AX expression in intestine after radiation at 3, 5 and 7 days. Error bars represent SD, *P < 0.05; **P < 0.01; ****P < 0.000, based on Student’s *t* test. (**I**, **J**) Western blot and statistical analysis of CUL4B, BRCA1 and p-BRCA1 expression in intestine after radiation at 3, 5 and 7 days. Error bars represent SD, **P < 0.01; ****P < 0.0001, based on Student’s *t* test. (**K**, **L**) Western blot and statistical analysis of CUL4B and RAD51 expression in intestine at 3 days post IR. Error bars represent SD, **P < 0.01; ****P < 0.0001, based on Student’s *t* test. The original western blots are presented in Fig. S6.

We have shown that CUL4B inhibits ubiquitination of PSME3 and thus impedes p53 protein expression in unirradiated mice. However, following radiation treatment, CUL4B no longer bound to PSME3 and deficiency of CUL4B not hinders but assists with intestinal recovery from radiation-induced damage, suggesting that CUL4B exerts a different mechanism during damage repair. It has been reported that nuclear localization of CUL4B is critical for its regulation of cell proliferation, so we speculate that CUL4B plays a different functional role after experiencing damage possibly due to its translocation in cells. Therefore, we detected the subcellular localization of CUL4B protein through immunofluorescence and nucleoplasm separation. At 3 days post-irradiation, a markedly increased percentage of nuclear importation of CUL4B was detected (Fig. 6A, B). More importantly, by measuring CUL4B in separated nucleoplasm, we found that CUL4B protein bound to chromatin was significantly elevated after irradiation (Fig. 6C, D), suggesting that CUL4B might enter the nucleus and positioned at the site of DNA damage after irradiation.

γH2AX, an indicator of DNA damage induced by radiation and the loss rate of which correlated with the rate of DBS repair^23^. To confirm whether CUL4B interacts with γH2AX and drives DNA damage, we carried out the immunoprecipitation and detected the clear combination between CUL4B and γH2AX (Fig. 6E, F). The colocalization of them in nucleus was evidenced in Fig. S4. Meanwhile, CUL4B deletion decreased γH2AX expression in intestine of mice and IEC-6 cells after irradiation treatment, confirming the correlation of CUL4B and γH2AX (Fig. 6G, H, Fig. S5A-S5B, Fig. S5G-S5H). Moreover, BRCA1 (p-BRCA1) and RAD51, DDR-related proteins recruited by γH2AX at damage lesions, were assayed by Western blot and immunofluorescence. We found that absence of CUL4B resulted in significantly elevated in p-BRCA1 expression and obviously increased RAD51 foci (Fig. 6I-L, Fig. S5C-S5H), further validating loss of CUL4B alleviates DNA damage.

**Figure 7:**
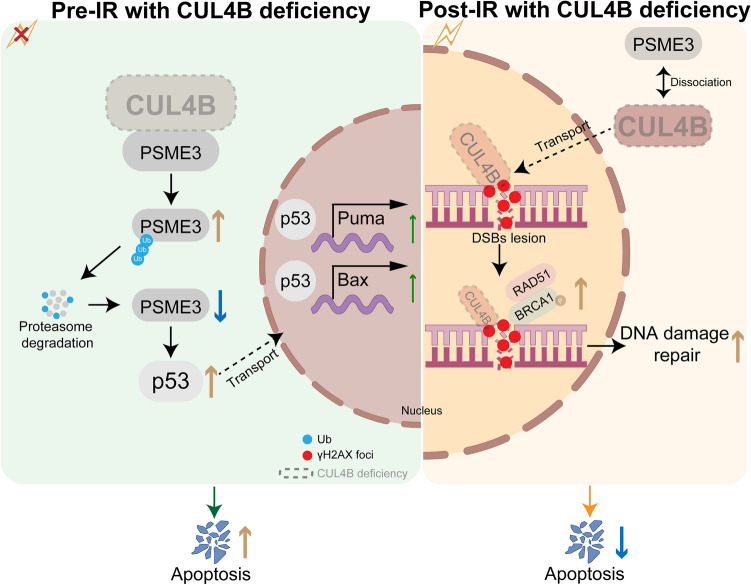
CUL4B plays a dynamic role in p53-mediated cell apoptosis during IR-induced intestine damage repair.

Taken together, our findings suggest that while CUL4B can inhibit p53-mediated apoptosis by coordinating and regulating PSME3 before irradiation. After irradiation, CUL4B dissociated from PSME3 and located at γH2AX foci in nuclear. On one hand, CUL4B deficiency diminished PSME3 expression and upregulated p53-mediated apoptosis upon damage. On the other hand, loss of nuclear CUL4B ultimately accelerated the cellular repair of DNA damage (Fig. 7).

## Discussion

The dependence of p53 signals characterizes IR-induced damage to the epithelium of small intestine^24,25^, while more regulators not fully understood. In this study, CUL4B was identified to play a promising role in the context of radiation-induced intestinal damage and recovery depending on p53 regulation, strengthening the comprehension of CUL4B in modulating impaired intestinal functions. Here we demonstrated the flexible and reversed function of CUL4B delimited by IR exposure. Prior to IR, CUL4B encouraged the expression of PSME3 through binding with PSME3 and inhibiting its degradation after ubiquitination. p53 was subsequently repressed by CUL4B-mediated PSME3. Post IR, CUL4B dissociated from PSME3, translocated into nucleus and colocalized at DNA damage sites, impeding DNA repair by down-regulating p-BRCA1 and RAD51. Our findings suggest a novel function of CUL4B in repair IR-induced damage in intestinal tissues.

It has been reported that CUL4B-DDB1 (DNA damage-binding protein 1) targeted to UV-damage chromatin and initiated efficient nucleotide excision repair (NER)^17^. Here we examined γH2AX as recognition substrate of CUL4B at the site of radiation lesions. The fact of high efficiency of monoubiquitinated histone H2A by CUL4B provides evidence for the above result^17,26^. The identification of subcellular localization of CUL4B is based on the its specific cytoplasm location in intestine cells^15^. CUL4B can be transported into the nucleus after radiation treatment to participate the DDR, due to its nuclear localization signal (NLS)^27^. This abrupt change indicates CUL4B will certainly play assignable role, and other related functions need to be further detected apart from DDR.

The occurrence of cell apoptosis is the consequence of redundancy DNA damage after radiation, in which p53 is a key factor and affected by multiple upstream signals. Nevertheless, it is worth noting the controversial role of p53 in cell specificity. p53 deficiency blocks apoptosis of most intestine epithelial cells and crypt cells rather than endothelial cells^24,28–30^. Undeniably, all findings in this study based on the comparison with and without CUL4B deletion in the intestinal epithelium, revealing CUL4B as a novel regulator of p53 in IR-induced apoptosis. In addition, a contrasting perspective has emerged in the function of p53, which prevents genotoxicity caused by IR through inducing revival and decreasing apoptosis of stem cells to reinstate intestine integrity^31,32^. Whether the protective property of p53 is related to CUL4B and various effects of downstream molecules of p53 seem to be further detected. Therefore, the alternate exact mechanism by which CUL4B regulates p53 needs further verification.

PSME3 is a member of the 11S proteasome activator, which binds and activates the 20S proteasome in a ubiquitin and ATP-independent manner^21^. Not only that, PSME3 is required in DDR and maintenance of centrosome and chromosomal stability^33,34^. Its exhaustion leads to cell radiosensitivity and significant delay in DSB repair^34^. Moreover, PSME3 is implicated in the existence of p53 in multiple levels. PSME3 facilitates the physical interaction of MDM2-p53 in the form of polymers, promoting the polyubiquitination of p53, subsequent nuclear export and MDM2-dependent proteasomal degradation to make cells resistant to apoptosis^35,36^. In turn, p53 protein promoted PSME3 transcription, which can be inhibited by PSME3 itself thus forming a negative feedback loop^22^. We found that PSME3 acts as a dynamic binding substrate of CUL4B, providing strong support for the change in DNA damage and cell apoptosis with or without radiation. Before irradiation, the combination of CUL4B between PSME3 resulted in a decrease of its ubiquitination level and subsequent increased of its accumulation, reduced DNA damage and p53-induced cell apoptosis. After irradiation, CUL4B dissociated from PSME3 and entered nucleus. p53-induced cell apoptosis exhibited the opposite effect. However, in terms of DDR, sustained higher expression of γH2AX seems to play a dominant role instead of PSME3 controlled by CUL4B, suggesting that the impact on cellular and molecular effects still needs further investigation.

In conclusion, our findings demonstrate an inventive function of CUL4B during the intestine damage and recovery stage caused by radiation. CUL4B binds to PSME3 to inhibit the expression of p53 without any stimulation but abandons PSME3 to bound with γH2AX at the DNA damage sites on chromatin and down-regulates phosphorylation of the DNA repair protein BRCA1 and RAD51 after radiation. In addition, CUL4B has been reported to be overexpressed in a range of cancers and promotes carcinogenesis through regulating multiple cellular malignant behaviors^37–42^, suggesting that CUL4B can serve as a potential therapeutic target simultaneously for cancers and intestinal injury caused by radiotherapy of cancers.

## Material and methods

### Animals

*Pvillin*-*Cre* mice (strain: C57BL/6J, stock number: T0116) were procured from Model Animal Research Center (MARC). *Cul4b*^*WT*^ (*Pvillin-Cre*^*−/−*^; *Cul4b*^*fn/fn*^ or *Cul4b*^*fn/Y*^) and *Cul4b*^*ΔIEC*^ (*Pvillin-Cre*^+*/−*^; *Cul4b*^*fn/fn*^ or *Cul4b*^*fn/Y*^) were generated by cross-breeding. Genotyping PCR was performed according to the genotyping protocol and primers were listed in Table 1. We did not exclude mice during data statistical analysis, and the included mice were confirmed by testing knockout efficiency through Western Blot or Immunostaining before processed according to experimental requirements without excessive attrition. All animal experiments were performed with approval of the Animal Care and Use Committee of the School of Basic Medical Science, Shandong University (KYLL-2017(KS)-363). The study was conducted in accordance with relevant guidelines and regulations. The study complied with ARRIVE guideline (https://arriveguidelines.org). 12-week male mice were randomly chosen and anesthetized with 1% pentobarbital sodium (40 mg/kg) by intraperitoneal injection, and further exposed to whole-body γ-irradiation with a dose of 12 Gy in a single dose using a Precision X-Ray 225 kV orthovoltage unit for intestinal injury model. The mice were sacrificed by cervical dislocation and small intestine were excised for processing at 0, 1, 3, 5 and 7 days after IR^43,44^.

**Table 1:** Primer sequence for genotyping.

	Sequence (5′–3′)
*Cre* forward primer	CCCGCAGAACCTGAAGATG
*Cre* reverse primer	GACCCGGCAAAACAGGTAG
*Loxp* forward primer	ACAGGTATTTGCCAGTGCTGTC
*Loxp* reverse primer	TTCTGTTACCTTCCTACCGAGAG

### Immunostaining

The small intestine was fixed in 4% paraformaldehyde, embedded in paraffin, and sectioned with a thickness of 4 μm. Some slides were stained with H&E for assessing intestine morphology. Some slides were cooked in 0.01 mol/L sodium citrate solution (pH 6.0) for 15 min, and incubated with 3% H_2_O_2_ or 0.5% TritonX-100, and blocked with 10% goat serum, and incubated with primary antibody over night at 4 °C followed by HRP-conjugated or fluorescent-tagged secondary antibody. Proximity Ligation Assay (PLA) was performed according to Duolink in Situ PLA (Sigma-Aldrich) protocol. Anti-Ki67 (Abcam, ab15580), anti-CUL4B (Sigma-Aldrich, Cat# C9995) and anti-PSME3 (Cell Signaling Technology, 95128S) were used.

IEC-6 cells were digested and added onto the sterile microscope slides pre-treated with polylysine at 5000 cells per well of 24-well plate. After 24 h post IR, the slides were washed by PBS and fixed with Immunol Staining Fix Solution (Beyotime, P0098) for 30 min. And then, the slides were treated with 0.5% TritonX-100, blocked with 10% goat serum for 1 h at room temperature, and incubated at 4 °C with primary antibodies overnight, followed by secondary antibodies. Anti-CUL4B (Sigma-Aldrich, Cat# C9995), anti-RAD51 (Abcam, ab133534) and anti-γ-H2AX (EMD Milipore, 05-636-25μg) were used as primary antibodies. Anti-rabbit Rhodamine (Jackson ImmunoResearch, 111-025-003) and anti-mouse Fluorescein (Jackson ImmunoResearch, 115-095-003) were used as secondary antibodies. Images were captured with OLYMPUS BX51.

### Cell culture and manipulation

IEC-6 was procured from Nanjing Foksay Biotechnology Co., Ltd. IEC-6 was cultured in DMEM (Gibco, 11965167) with 10% fetal bovine serum (Gibco, 10099-141C), penicillin (100 mg/mL, BBI, A600135) and streptomycin (100 mg/mL, BBI, A100382) at 37 °C in a humidified atmosphere (5% CO_2_). The cell line was identified and tested for Myco-Blue Mycoplasma Detector (Vazyme, D101-02). *Cul4b* knockdown (sh*Cul4b*, 5′-AATATTTCCCGGAACATTCTG-3′) cells and control cells (shNC) were generated by infecting cells with lentivirus^42^. Briefly, cells were transfected with virus after 24 h plated in 6-mm dishes, and changed into fresh culture medium after 24 h post transfection, and selected by puromycin (Gibco, A1113803). All cell lines were radiated with a dose rate of 3 Gy and collected at 0, 12, 24, 36 and 48 h after IR.

For protein stability assay, sh*Cul4b* and shNC were transfected with Psme3-expression plasmid and treated with cycloheximide (CHX, MCE, HY-12320, 120 μg/mL) to inhibit protein synthesis and collected at 0, 8, 16 and 24 h for immunoblotting.

For ubiquitination assay, IEC-6 were transfected with indicated plasmids or siRNAs (GenePharma Co., Ltd) and treated with MG132 (MCE, HY-13259, 20 μM). Cells were harvested with PBS containing NEM (MCE, HY-D0843S, 10 μM) to prevent deubiquitination, and then lysed in cell lysis buffer containing Tris 8.0 (10 mM), NaCl (150 mM) and 2% SDS by boiling at 95 °C for 10 min, followed by sonication (8 s, 1 cycle). Lysed sample supernatants were then incubated with anti-PSME3, mixed with protein A/G PLUS-Agarose (Santa Cruz, sc-2003) for 12 h at 4 °C, followed by Western blot.

### Flow cytometry analysis

For analysis of apoptosis, IEC-6 cells were fixed and stained with PE Annexin V Apoptosis Detection kit with 7-AAD (Vazyme, A213-01) according to the manufacturer’s instructions. The proportion of PI-positive and PE-positive cells were recorded. Data acquisition was performed on Accuri C6 Plus and analyzed via FlowJo (RRID:SCR_008520).

### RNA isolation and RT-qPCR

For RNA isolation, intestine tissues were lysed in Trizol (Invitrogen, 15596018) and reversely transcribed with HiScript III RT SuperMix for qPCR (Vazyme, R323). RT-qPCR analysis was performed with SYBR Green Mixture (Roche, 4729692001) in qTOWER3G. The primers for RT-qPCR were designed using Primer3 (RRID:SCR_003139) and BLAST through NCBI and listed in Table 2. Gene expression was normalized to the referee *Gapdh* or *Rps18* to control.

**Table 2:** Primers for RT-qPCR.

	Sequence (5′–3′)
*m-Gapdh* forward	AGGTCGGTGTGAACGGATTTG
*m-Gapdh* reverse	TGTAGACCATGTAGTTGAGGTCA
*m-Puma* forward	ATGAGCCAAACCTGACCACT
*m-Puma* reverse	TGAGATGGATGGGGATTGGG
*m-Bax* forward	AGACAGGGGCCTTTTTGCTAC
*m-Bax* reverse	AATTCGCCGGAGACACTCG
*m-Cul4b* forward	TGGAAGTTCATTTACCACCAGAGATG
*m-Cul4b* reverse	TTCTGCTTTTAACACACAGTGTCCTA
*m-Il6* forward	CCAAGAGGTGAGTGCTTCCC
*m-Il6* reverse	CTGTTGTTCAGACTCTCTCCCT
*m-Ilβ* forward	GCCCATCCTCTGTGACTCAT
*m-Ilβ* reverse	AGGCCACAGGTATTTTGTCG
*m-Tnfα* forward	GACGTGGAACTGGCAGAAGAG
*m-Tnfα* reverse	TTGGTGGTTTGTGAGTGTGAG
*m-Ifnβ* forward	CAGCTCCAAGAAAGGACGAAC
*m-Ifnβ* reverse	GGCAGTGTAACTCTTCTGCAT
*Rat-Rps18* forward	ATCCCCGAGAAGTTTCAGCA
*Rat-Rps18* reverse	ATTGTCGTGGGTTCTGCATG
*Rat-Puma* forward	AAACCTGACCACTAGCCTCC
*Rat-Puma* reverse	AATGGGATGGATGGGGACTG
*Rat-Bax* forward	GAGACACCTGAGCTGACCTT
*Rat-Bax* reverse	CGTCTGCAAACATGTCAGCT
*Rat-Cul4b* forward	TTAAGGAACGGGTGGCTGAT
*Rat-Cul4b* reverse	AATCTCTGGGTTGTGGAGGG

### Western blot

Total proteins were extracted with Trizol, protein lysis or Subcellular Protein Fractionation Kit for Tissues (Thermo, 87790) following the manufacturer’s instructions. A total of 40 μg of protein were separated on a 10% SDS-PAGE and transferred onto a PVDF membrane. The membranes were blocked with 5% fat-free milk in Tris-buffered saline (TBS) containing 0.1% Tween-20 (BBI, 9005-64-51) for 1 h at room temperature and then incubated with primary antibodies against CUL4B (Sigma-Aldrich, Cat# C9995), GAPDH (Cell Signaling Technology, Cat# 5174, RRID:AB_10622025), ACTIN (Santa Cruz, sc-69879), p-TBK1 (Ser172, Cell Signaling Technology, Cat# 5483), STING (p-STING, Cell Signaling Technology, Cat# 50494), p53 (Santa Cruz, sc-126), Caspase3 (Proteintech, 19677–1-AP), Cleaved-Caspase3 (Cell Signaling Technology, Cat# 9661), BAX (Cell Signaling Technology, 2772), PSME3 (Cell Signaling Technology, 95128S), γ-H2AX (EMD Milipore, 05-636-25ug), HSP90 (Cell Signaling Technology, C45G5), HDAC2 (Cell Signaling Technology, 57156), BRCA1 (Cell Signaling Technology, 9010T), p-BRCA1 (Ser1524, Cell Signaling Technology, 9009T) and RAD51 (Abcam, ab133534) overnight at 4 °C. The membranes were washed in TBST and incubated with HRP-labeled secondary antibody (anti-rabbit: Jackson ImmunoResearch, 111-005-003; anti-mouse: Jackson ImmunoResearch, 115-005-003) for 1 h at room temperature. The blots were cut prior to hybridisation with antibodies during blotting. After incubation, membranes were washed and proteins were detected using a SuperSignal Chemiluminescence kit (Thermo, 34580).

### Immunoprecipitation

Protein supernatants were incubated with indicated antibodies and protein A/G PLUS-Agarose (Santa Cruz, sc-2003) for 2 h at 4 °C. Immunoprecipitates were boiled in sample loading dye at 95 °C for 5 min, followed by Western blotting.

### EdU assay

EdU staining of IEC-6 was performed with Cell-LightTM EdU Apollo 567 In Vitro Kit (RiboBio, C10310-1) following the manufacturer’s instructions. EdU medium (1:1000) was added when IEC-6 cells were passaged and incubated for 2 h at 37 °C in a humidified chamber with 5% CO_2_. Reaction mix was added to label EdU after cells were fixed and permeabilizated, and the images were captured with Olympus BX51.

### Statistical analysis

Experimental operation was performed with biological or independent replicates. Statistical analysis was performed using GraphPad Prism. Data significance was analyzed using unpaired, two-tailed Student's *t* test or Log-rank test. Bar graphs represented mean ± standard deviation (SD). *P* values * < 0.05, ** < 0.01, *** < 0.001, **** < 0.0001, *ns* not significant.

### Supplementary Information

Supplementary Figures.

Supplementary Table S1.

Supplementary Table S2.

Supplementary Table S3.

Supplementary Table S4.

Supplementary Table S5.

Supplementary Table S6.

## Data Availability

The mass spectrometry data in this study are provided in Table S1–S3 and other detailed data are available upon request from the huhuili@sdu.edu.cn.
