# Supplementary material for: Dynamic role of CUL4B in radiation-induced intestinal injury-regeneration
Source: Sci Rep. 2024 Apr 30;14:9906. doi: 10.1038/s41598-024-60704-4 (PMC11061312; doi:10.1038/s41598-024-60704-4)
Supplement: Supplementary file 1 — Supplementary Figures. [file 41598_2024_60704_MOESM1_ESM.pdf]

# Title: Dynamic role of CUL4B in radiation-induced intestinal injury-regeneration

Beibei Guo[1], Xiaohan Huo[1,2], Xueyong Xie[2], Xiaohui Zhang[1], Jiabei Lian[1], Xiyu Zhang[2], Yaoqin Gong[2], Hao Dou[2], Yujia Fan[1], Yunuo Mao[1], Jinshen Wang[3]*, Huili Hu[1]*

[1] The Key Laboratory of Experimental Teratology, Ministry of Education and Department of Systems Biomedicine and Research, School of Basic Medical Sciences, Cheeloo Medical College, Shandong University, 250012 Jinan, China.

[2] The Key Laboratory of Experimental Teratology, Ministry of Education and Department of Molecular Medicine and Genetics, Shandong University Cheeloo Medical College, School of Basic Medical Sciences, 250012 Jinan, China.

[3] Department of Gastrointestinal Surgery, Shandong Provincial Hospital Affiliated to Shandong First Medical University, Jinan, Shandong 250021.

**Corresponding Author**

* Huili Hu, School of Basic Medical Sciences, Shandong University, 250012 Jinan, China. Phone: +86-531-88382236; Fax: +86-531-88382236; E-mail: huhuili@sdu.edu.cn.

Jinshen Wang, Department of Gastrointestinal Surgery, Shandong Provincial Hospital Affiliated to Shandong First Medical University, Jinan, Shandong 250021; E-mail: doctorwjs@163.com.

## Supplementary Figures and Figure legends

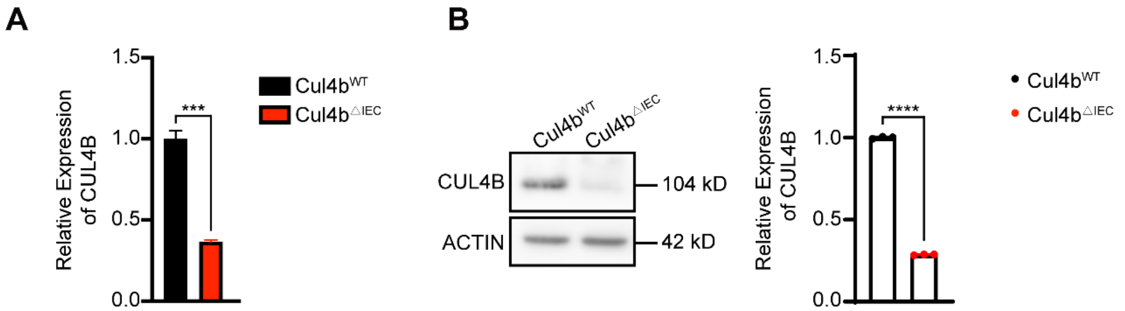


**Figure S1. Knockout efficiency of *Cul4b$^{ΔIEC}$* mice**

(A-B) Knockout efficiency of CUL4B were confirmed by RT-qPCR and Western blot in *Cul4b$^{ΔIEC}$* mice. Error bars represent SD, ****P*<0.001; *****P*<0.0001, based on Student's t test. The original western blots are presented in Fig S6.

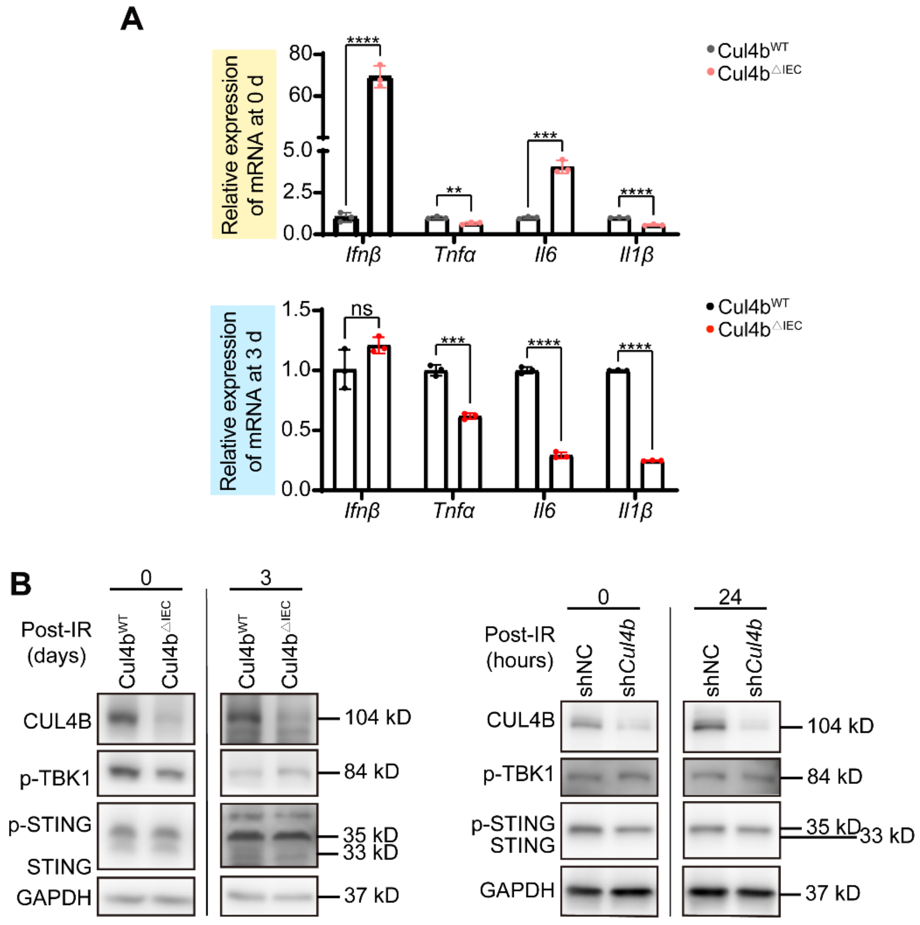


**Figure S2. Inflammation-associated effect was not obviously changed caused by the dynamic function of CUL4B after injury**

(A) RT-qPCR tested the effect gene of cGAS/STING pathway *Ifnβ*, and classic inflammation factors *Tnfα*, *Il6* and *Il1β* in vivo. Error bars represent SD, ***P*<0.01; ****P*<0.001; *****P*<0.0001; ns, not significant, based on Student's t test.

(B) Western blot and statistical analysis of the core proteins of cGAS/STING pathway in mice post IR at 0 and 3 days and in IEC-6 cells post IR at 0 and 24 hours.

The original western blots are presented in Fig S6.

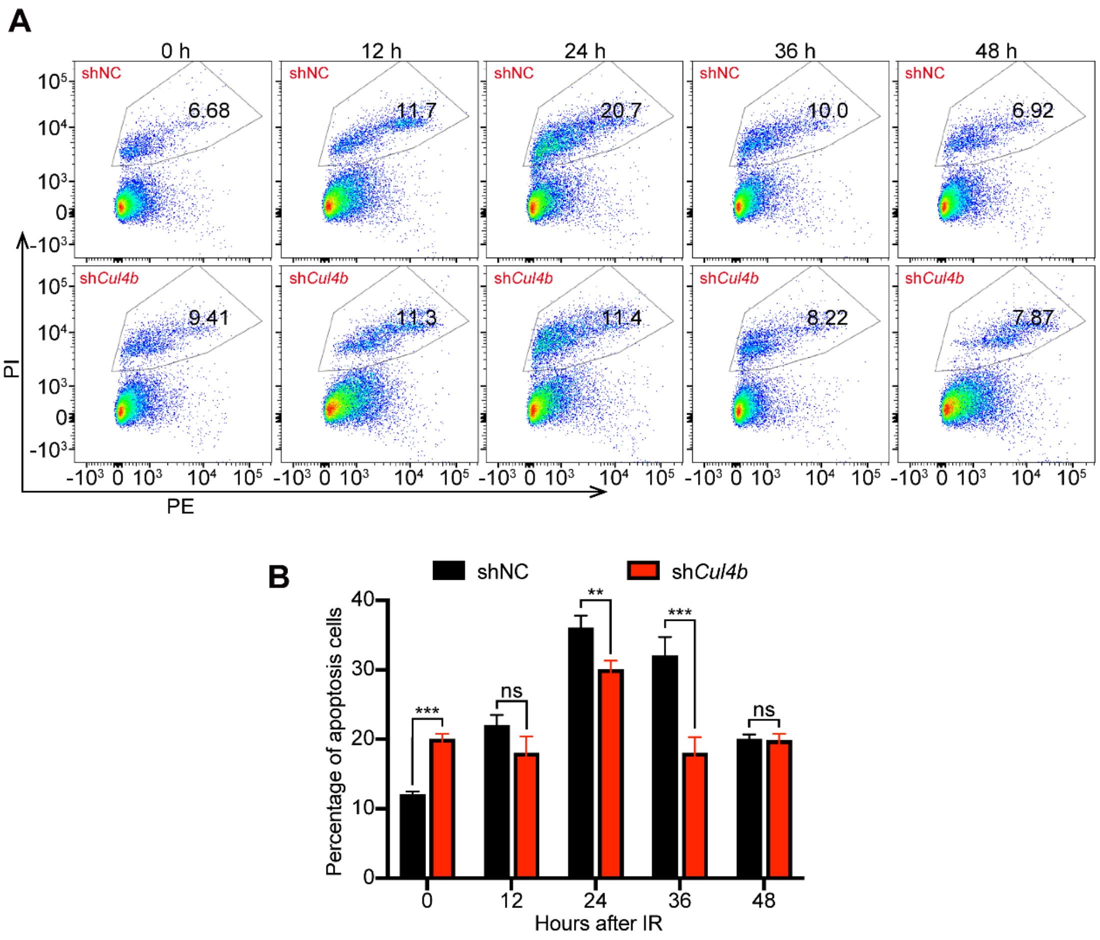


**Figure S3. Apoptosis was tested with IEC-6 cells**

(A-B) Flow cytometry analysis of apoptotic cells in sh*Cul4b* and shNC IEC-6 cells after radiation at 0, 12, 24, 36 and 48 hours. Error bars represent SD, ***P*<0.01; ****P*<0.001; ns, not significant, based on Student's t test.

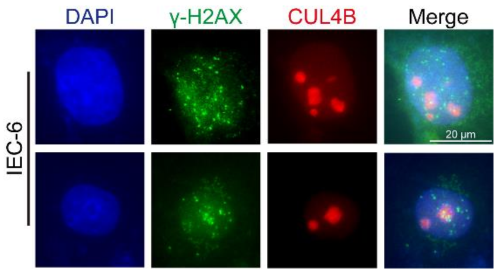


**Figure S4. The colocalization of CUL4B and γH2AX in nucleus in IEC-6 cells**

Representative images of immunofluorescence of γH2AX and CUL4B in IEC-6 cells post-IR at 24 hours.

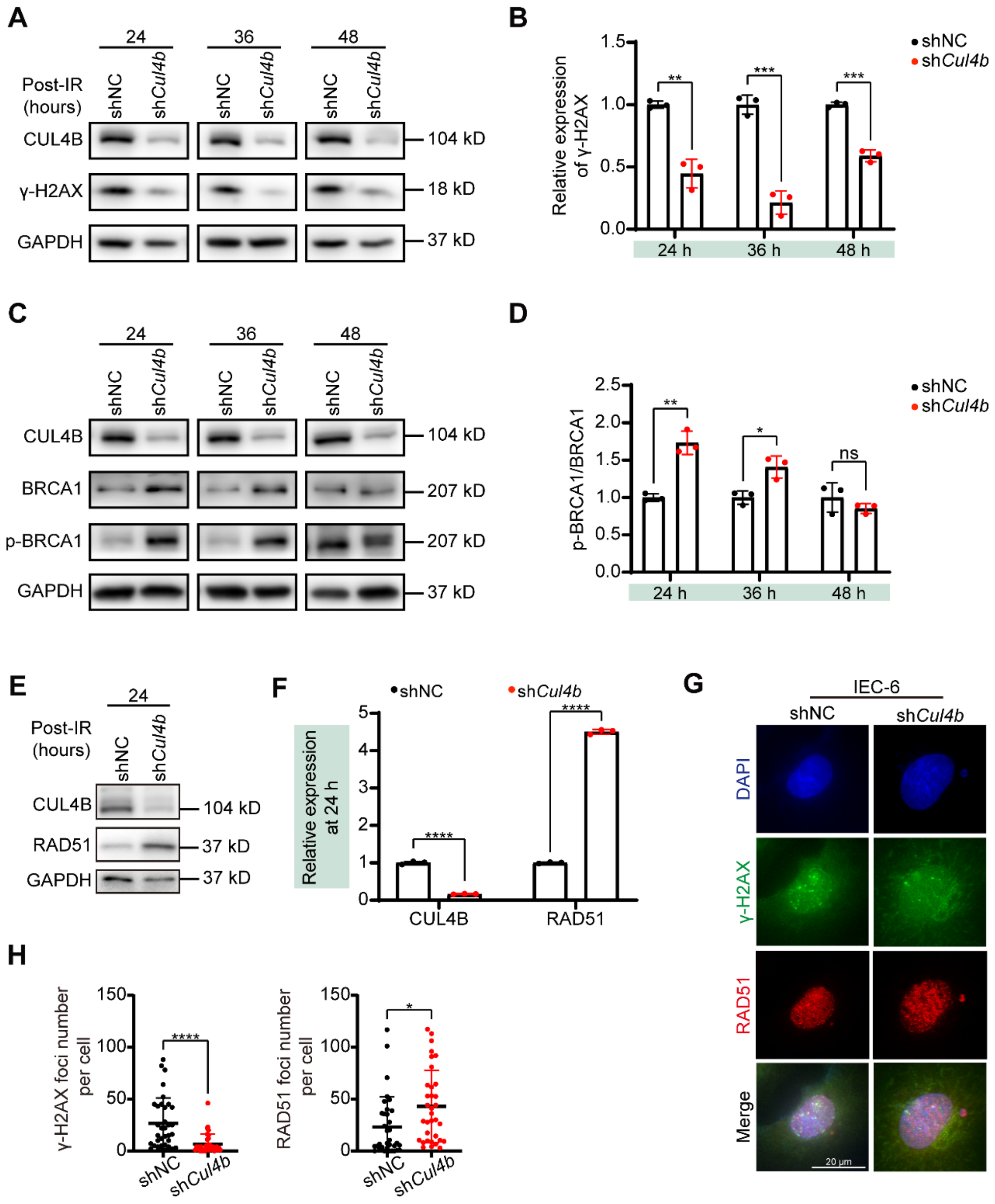


**Figure S5. CUL4B knockdown reduced DNA damage and enhanced repair in IEC-6 cells**

(A-B) Western blot and statistical analysis of CUL4B and γ-H2AX expression in sh*Cul4b* and shNC IEC-6 cells post-IR at 24, 36 and 48 hours. Error bars represent SD, ***P*<0.01; ****P*<0.001, based on Student's t test.

(C-D) Western blot and statistical analysis of CUL4B, BRCA1 and p-BRCA1 expression in sh*Cul4b* and shNC IEC-6 cells post-IR at 24, 36 and 48 hours. Error bars represent SD, **P*<0.05; ***P*<0.01; ns, not significant, based on Student's t test.

(E-F) Western blot and statistical analysis of CUL4B and RAD51 expression in sh*Cul4b* and shNC IEC-6 cells post-IR at 24 hours. Error bars represent SD, ****$P<0.0001$, based on Student's t test.

(G-H) Representative images and statistical analysis of immunofluorescence of γH2AX and RAD51 in sh*Cul4b* and shNC IEC-6 cells post-IR at 24 hours. Error bars represent SD, *$P<0.05$; ****$P<0.0001$, based on Student's t test.

The original western blots are presented in Fig S6.

Figure 1B

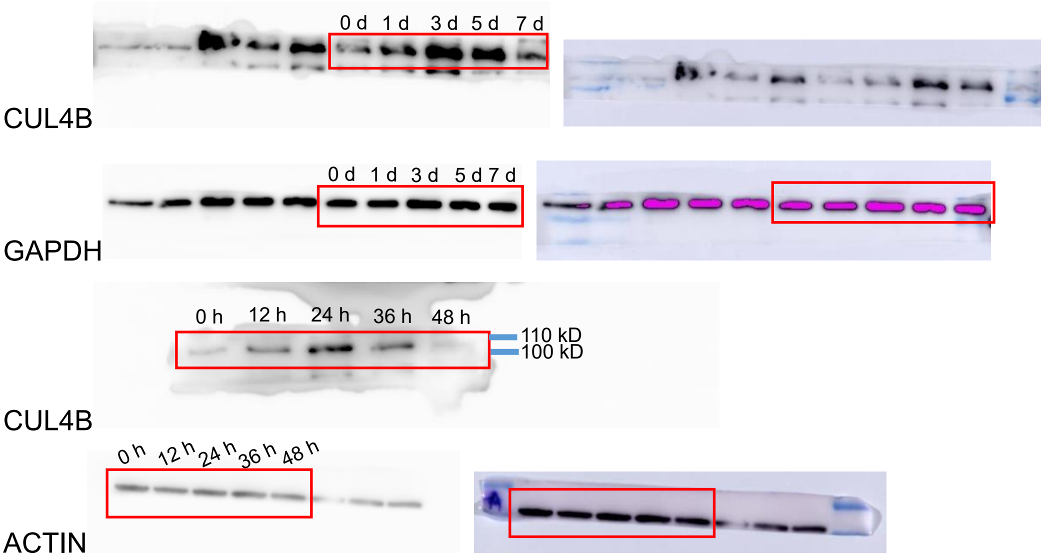
CUL4B
0 d 1 d 3 d 5 d 7 d
GAPDH
0 d 1 d 3 d 5 d 7 d
CUL4B
0 h 12 h 24 h 36 h 48 h
110 kD
100 kD
ACTIN
0 h 12 h 24 h 36 h 48 h

Figrue 2E (left panel)

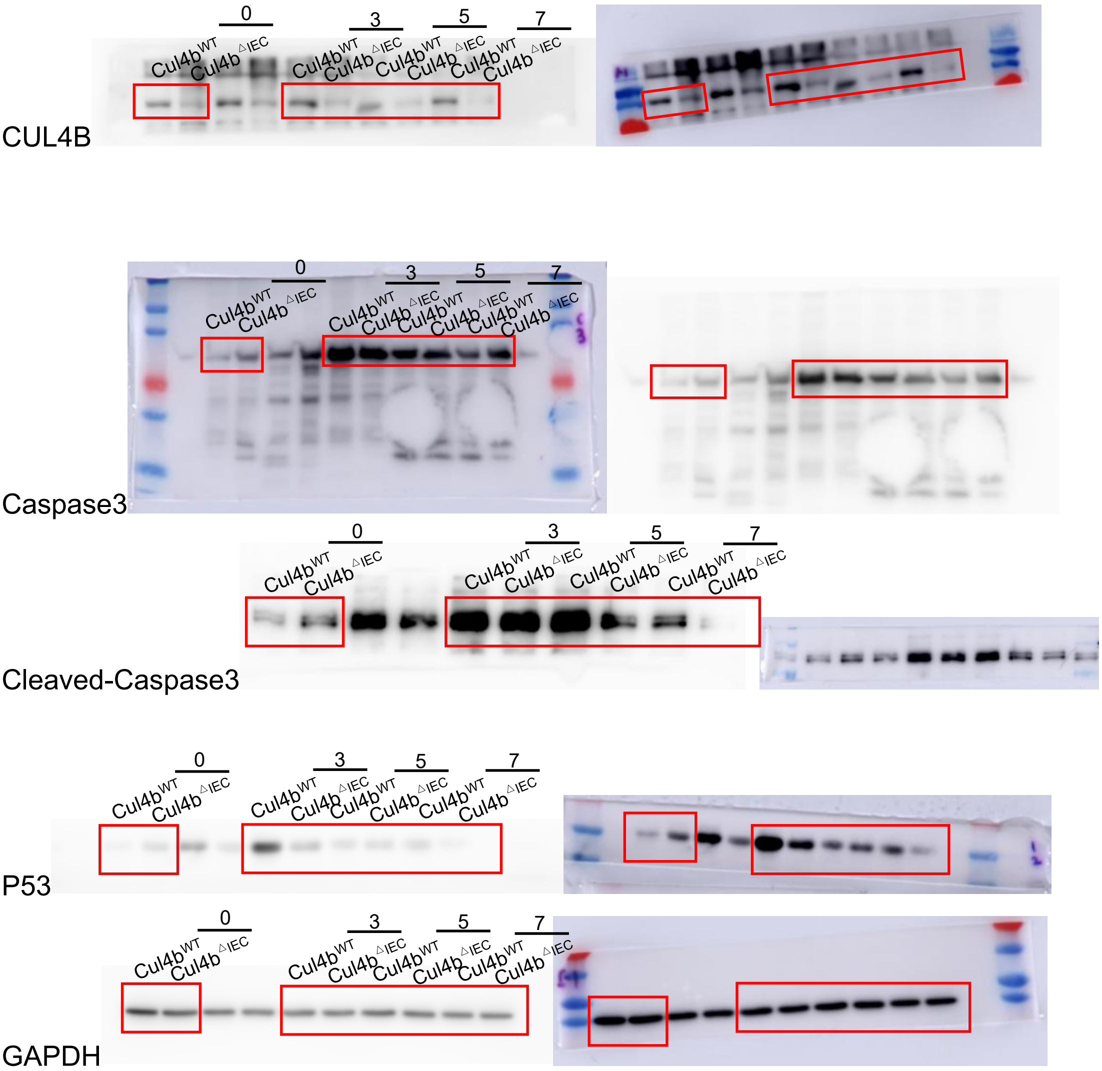

Figrue 2E (right panel)

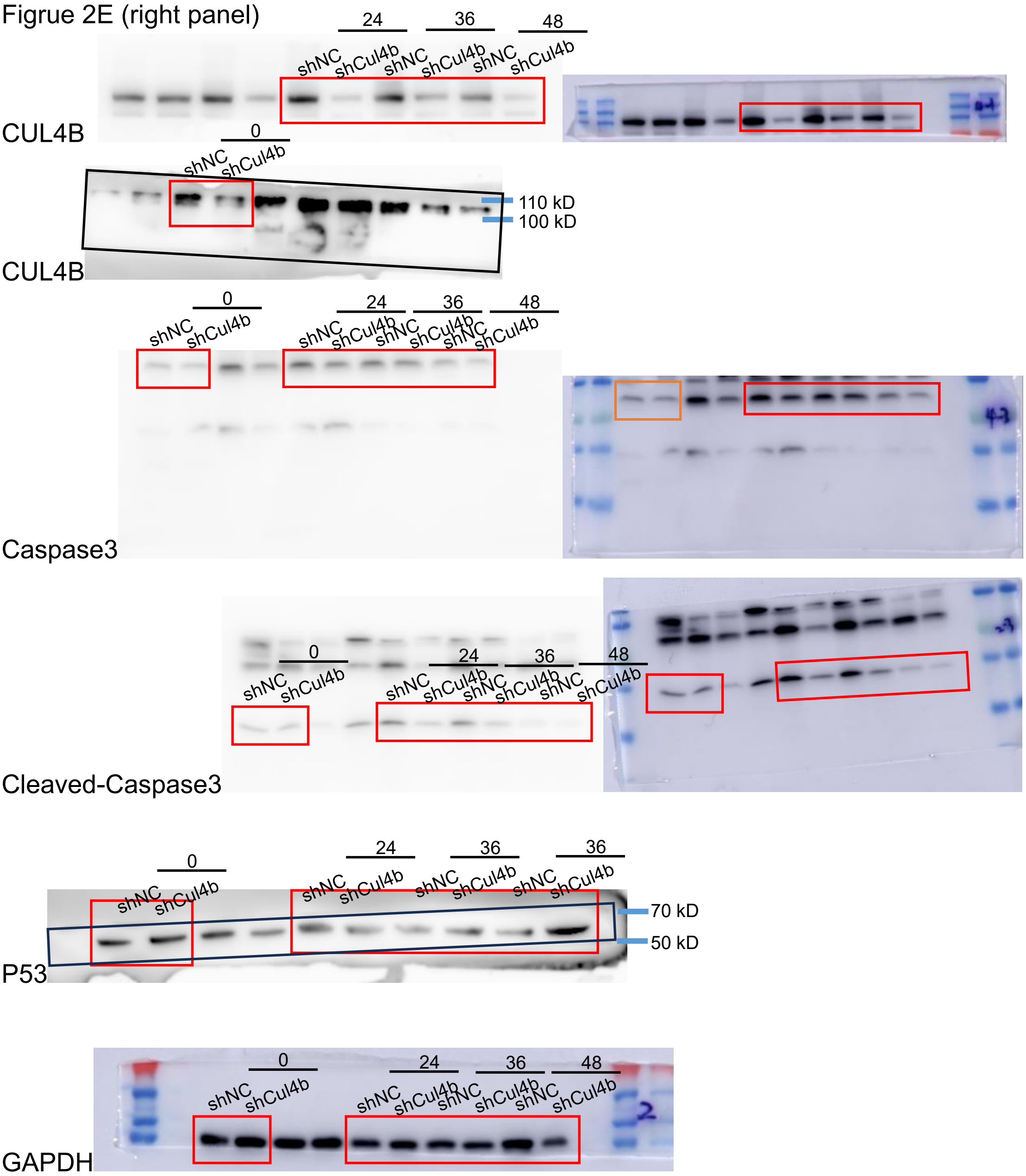

Figrue 3C

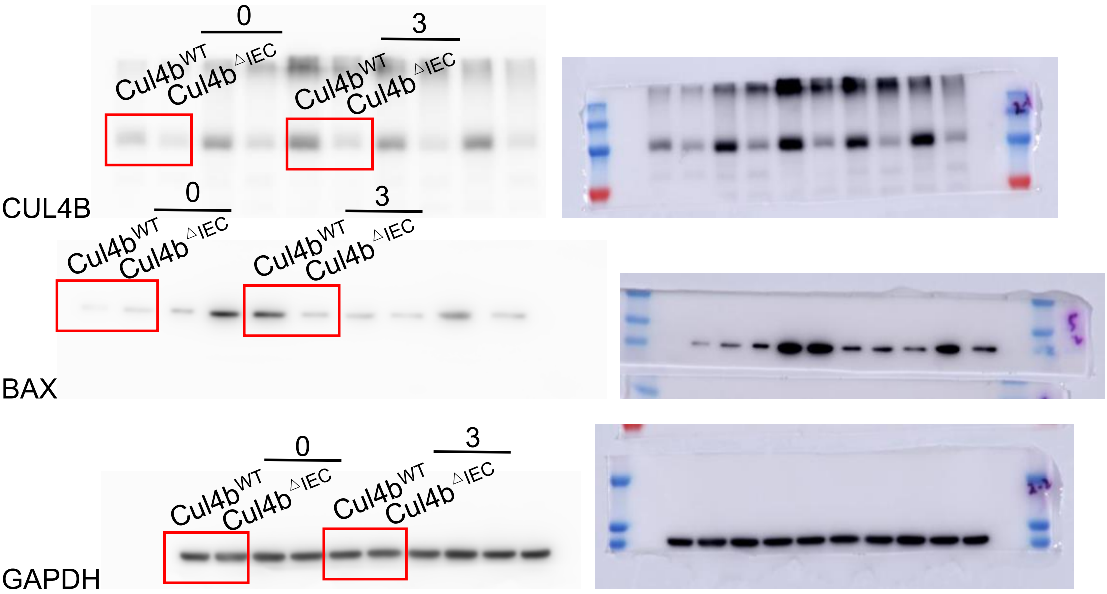

Figrue 3E

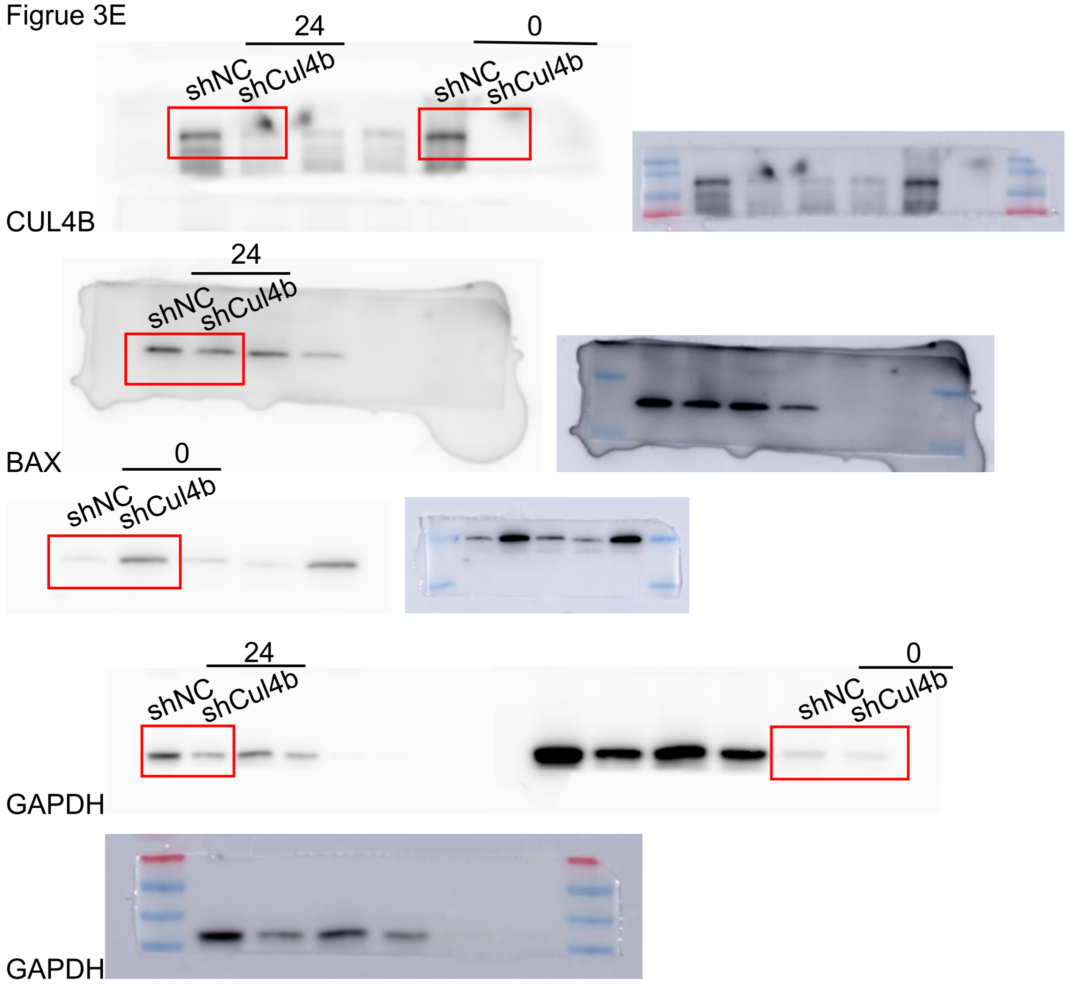
24
0
shNC
shCul4b
shNC
shCul4b
CUL4B
24
shNC
shCul4b
BAX
0
shNC
shCul4b
24
shNC
shCul4b
0
shNC
shCul4b
GAPDH
GAPDH

Figure 4D

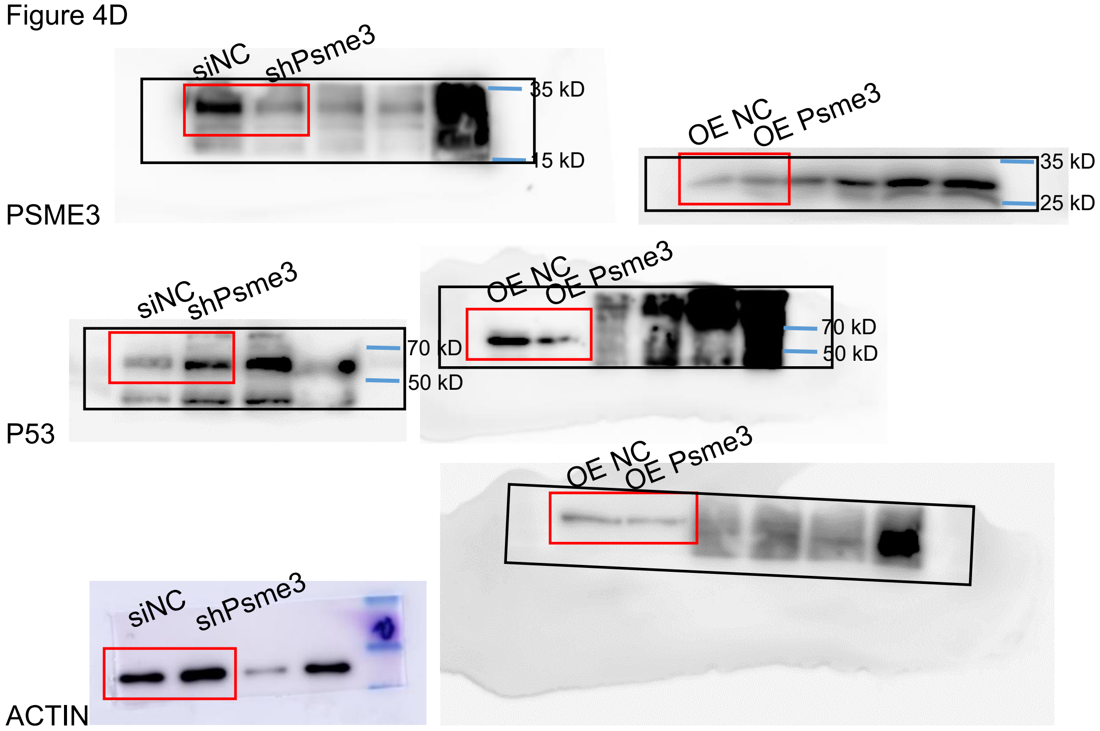
siNC
shPsme3
35 kD
15 kD
OE NC
OE Psme3
35 kD
25 kD
PSME3
siNC
shPsme3
70 kD
50 kD
OE NC
OE Psme3
70 kD
50 kD
P53
OE NC
OE Psme3
siNC
shPsme3
ACTIN

Figure 5A

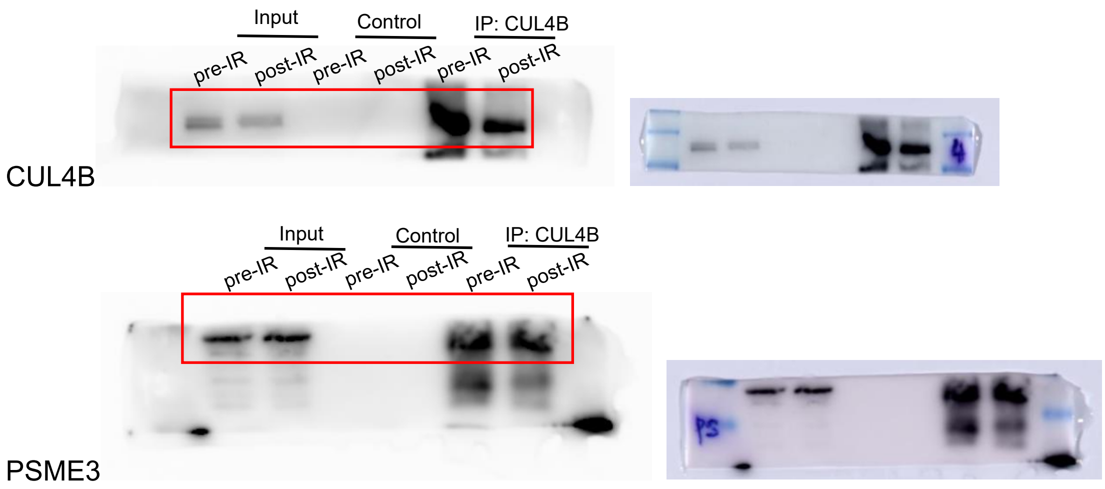
Input
Control
IP: CUL4B
pre-IR
post-IR
pre-IR
post-IR
pre-IR
post-IR
CUL4B
Input
Control
IP: CUL4B
pre-IR
post-IR
pre-IR
post-IR
pre-IR
post-IR
PSME3

Figure 5E

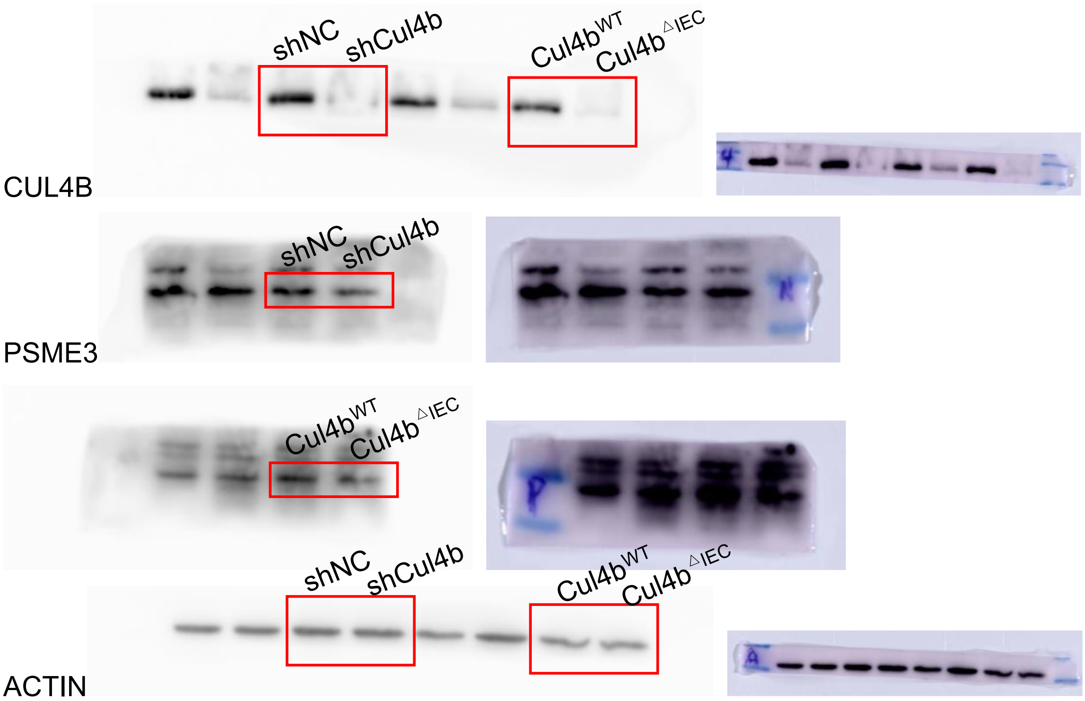

Figure 5G

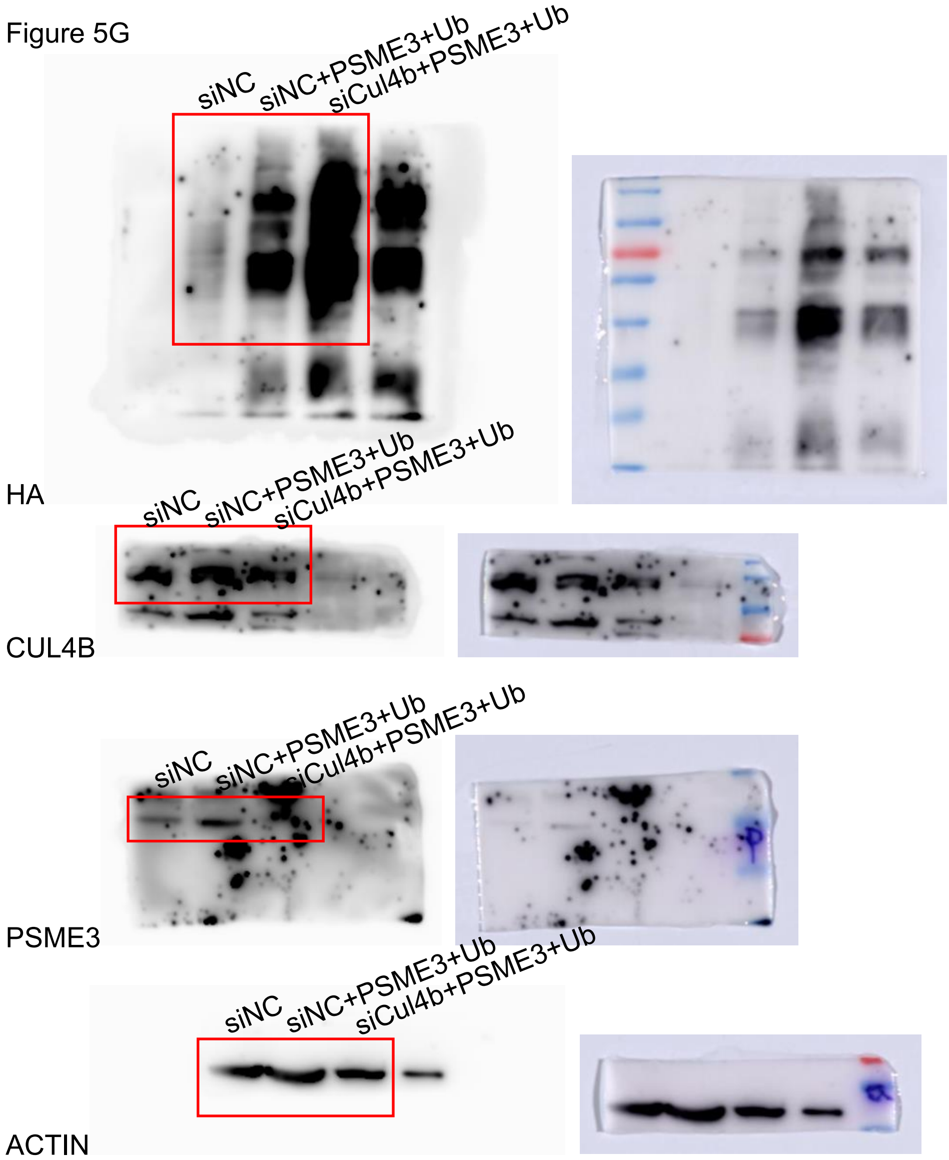

Figure 5H

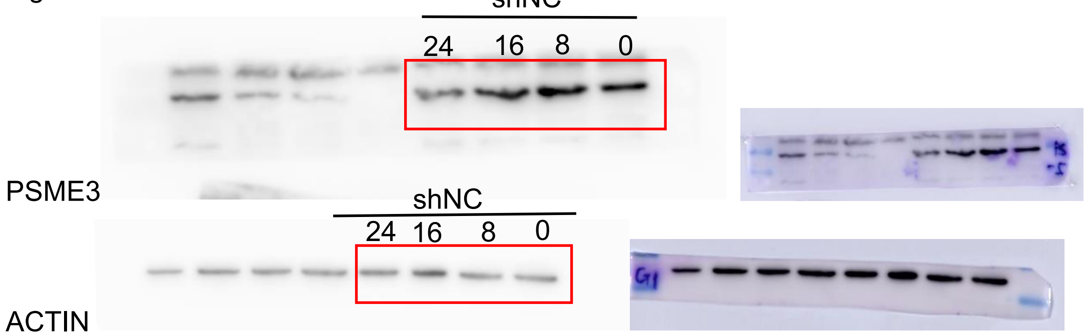


Above presented images were flipped due to the incorrect sample order when loading in Western blot.

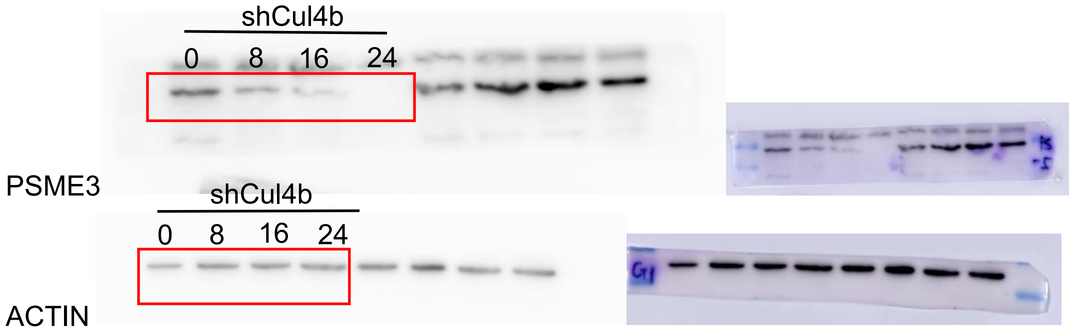

Figure 5J

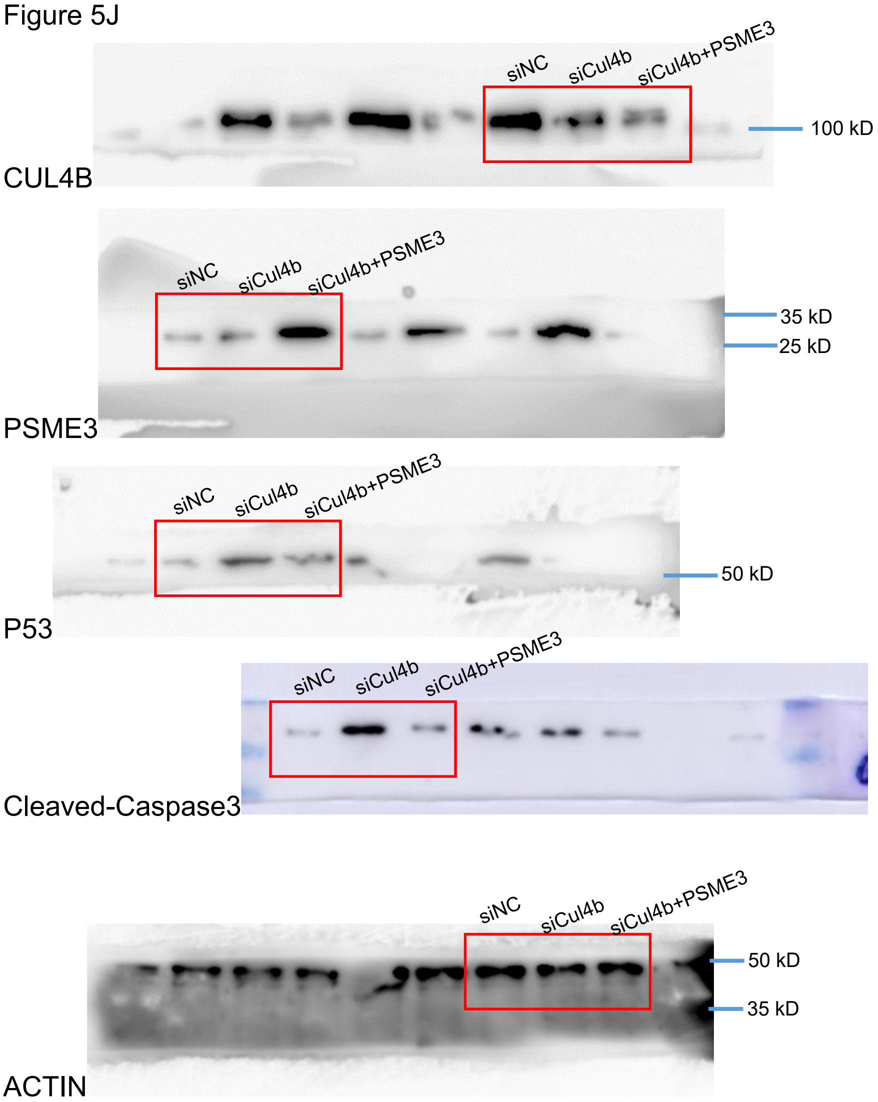
siNC
siCul4b
siCul4b+PSME3
100 kD
CUL4B
siNC
siCul4b
siCul4b+PSME3
35 kD
25 kD
PSME3
siNC
siCul4b
siCul4b+PSME3
50 kD
P53
siNC
siCul4b
siCul4b+PSME3
Cleaved-Caspase3
siNC
siCul4b
siCul4b+PSME3
50 kD
35 kD
ACTIN

Figure 6C

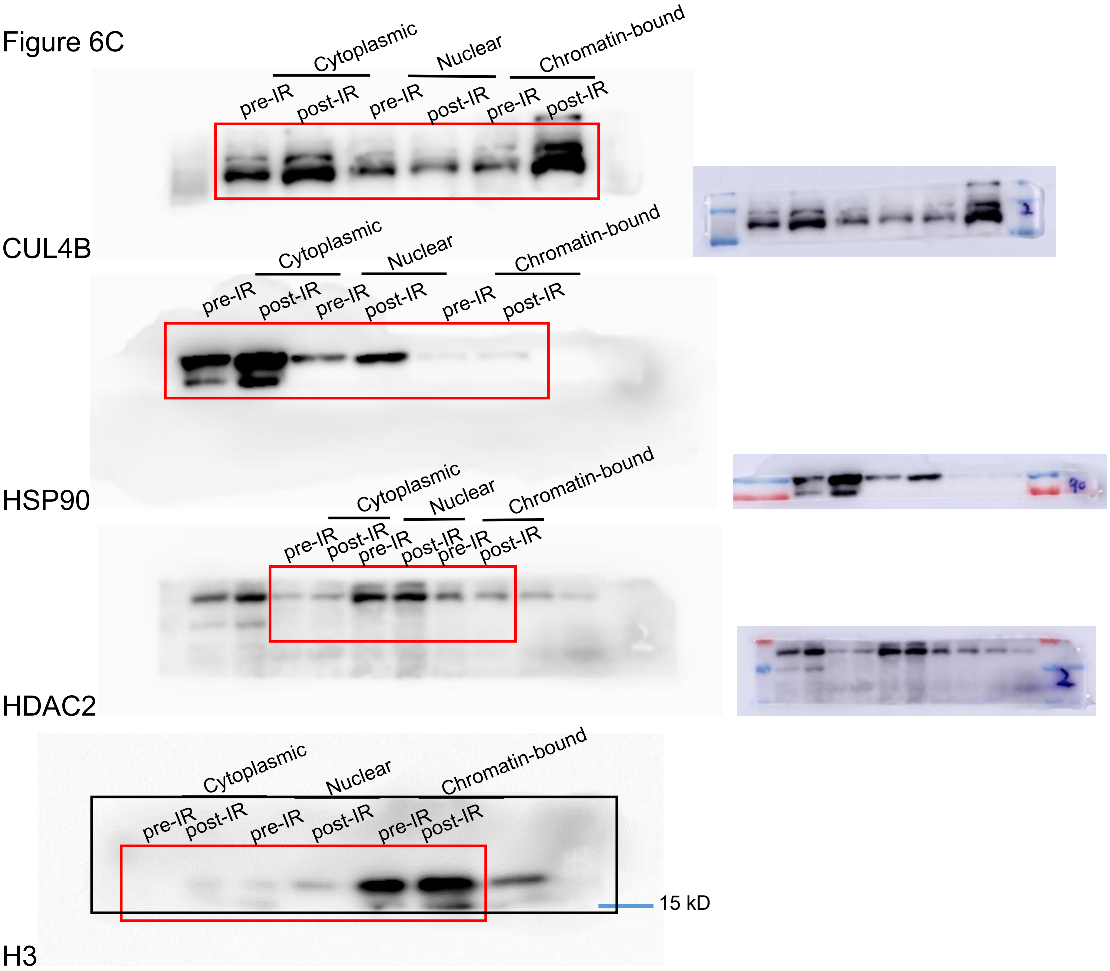
Cytoplasmic
Nuclear
Chromatin-bound
pre-IR
post-IR
pre-IR
post-IR
pre-IR
post-IR
CUL4B
HSP90
HDAC2
H3
15 kD

Figure 6E

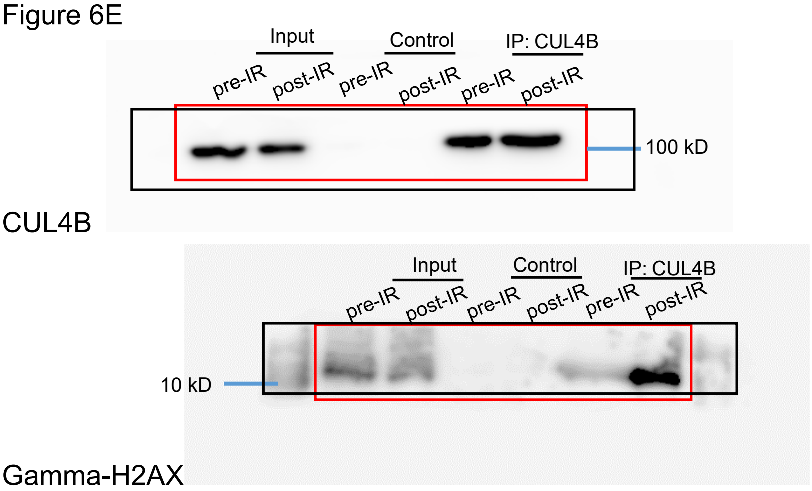
Input
Control
IP: CUL4B
pre-IR
post-IR
pre-IR
post-IR
pre-IR
post-IR
100 kD
CUL4B
Input
Control
IP: CUL4B
pre-IR
post-IR
pre-IR
post-IR
pre-IR
post-IR
10 kD
Gamma-H2AX

Figure 6G

CUL4B

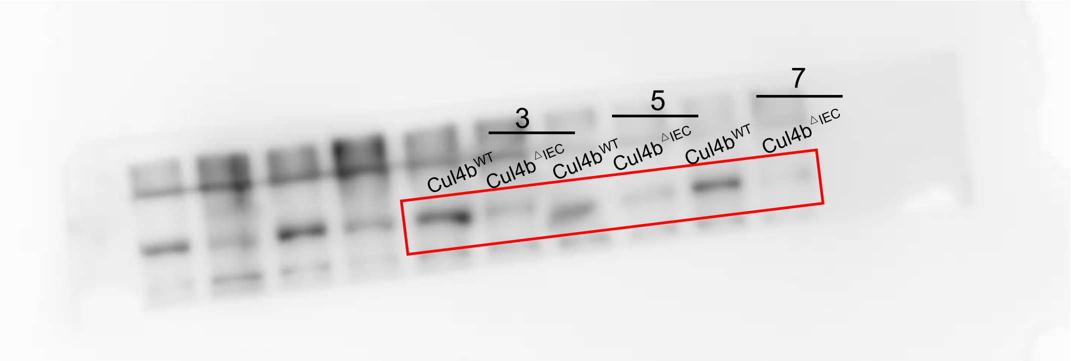


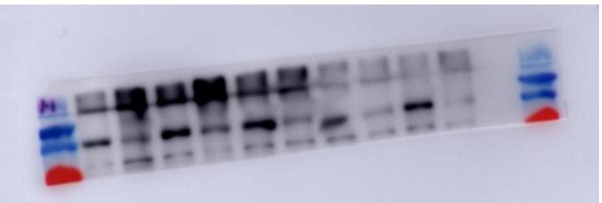

Gamma-H2AX

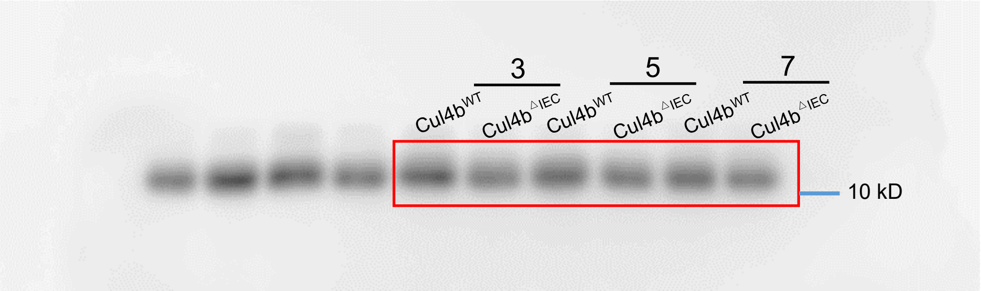


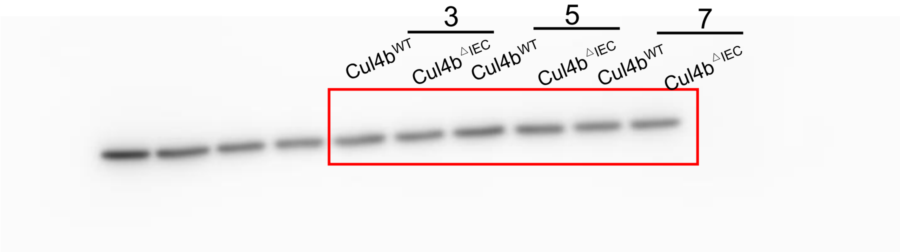


GAPDH

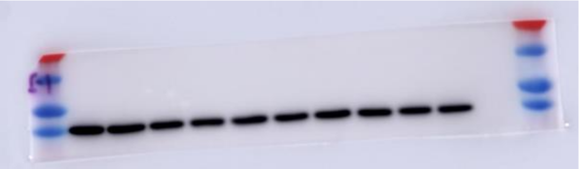

Figure 6I

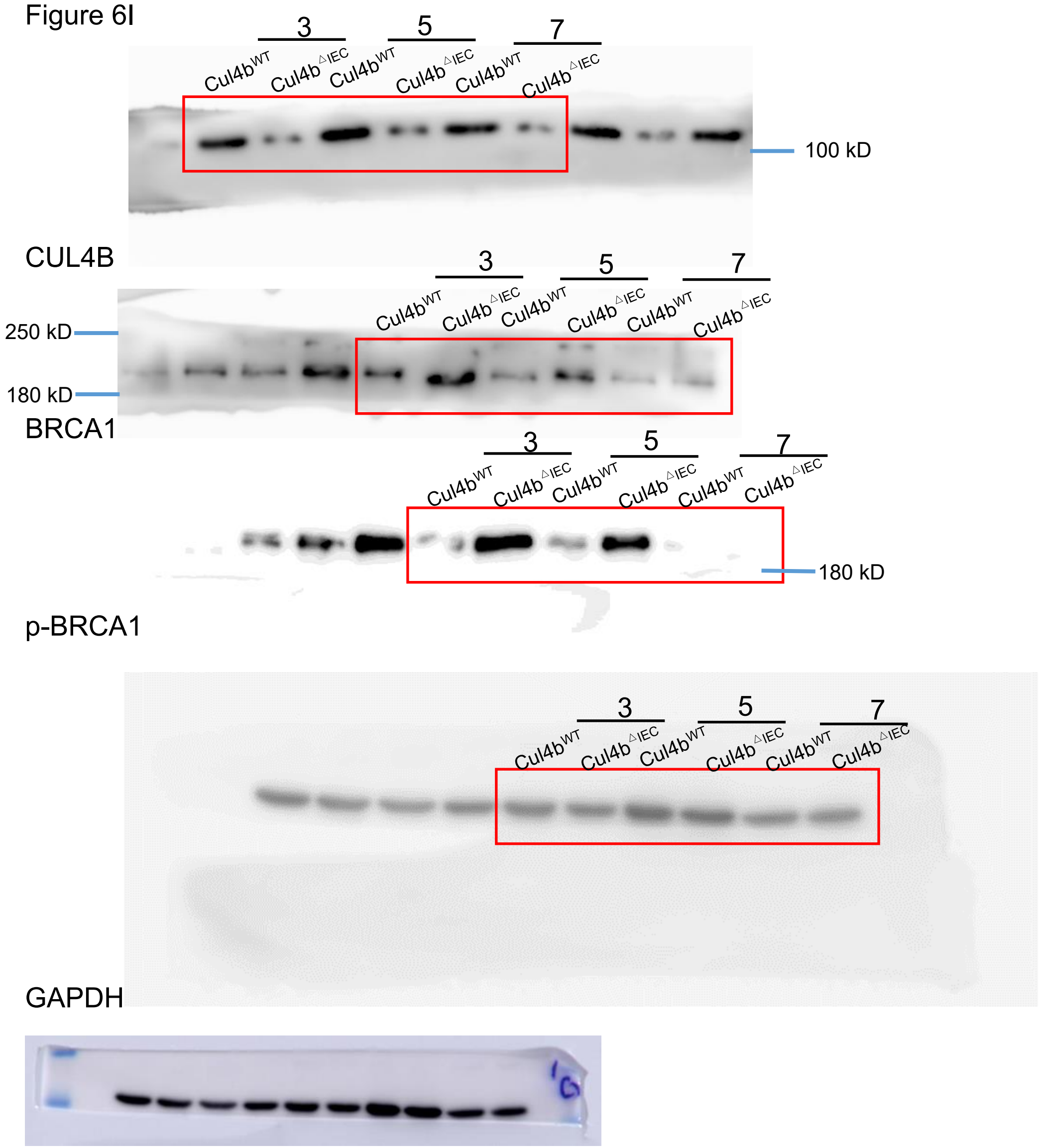

3
5
7
Cul4b$^{WT}$
Cul4b$^{\triangle IEC}$
Cul4b$^{WT}$
Cul4b$^{\triangle IEC}$
Cul4b$^{WT}$
Cul4b$^{\triangle IEC}$
100 kD
CUL4B
250 kD
180 kD
BRCA1
180 kD
p-BRCA1
GAPDH

Figure 6K

CUL4B

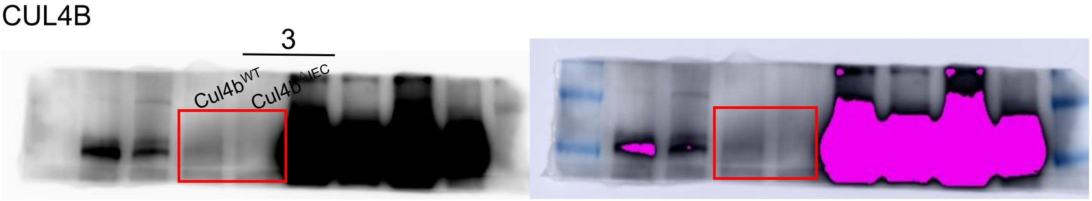


RAD51

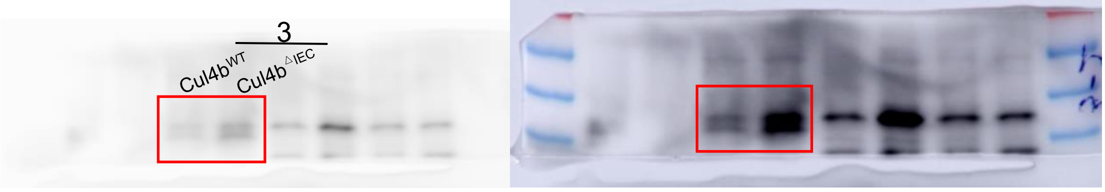


GAPDH

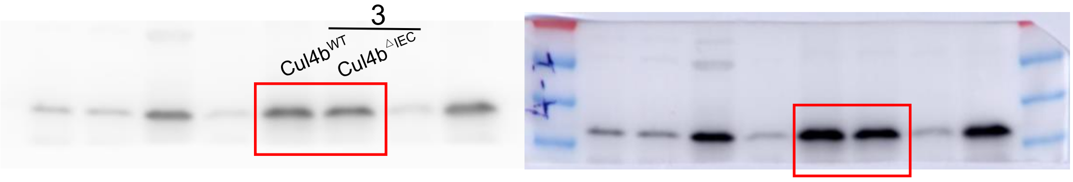

Figure S1B

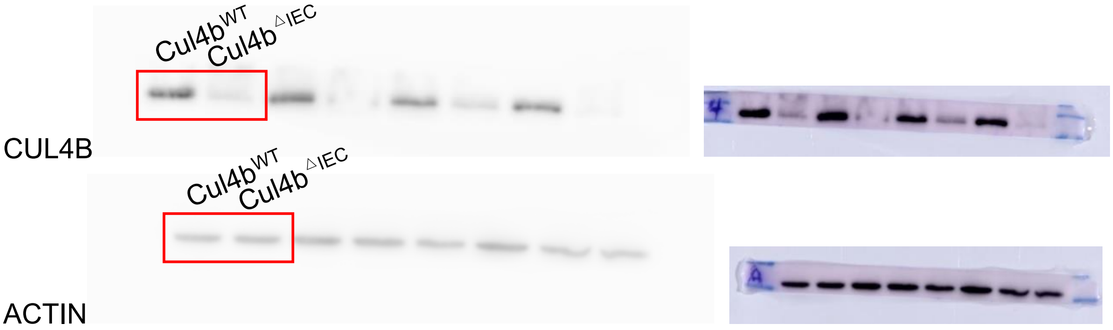

Figure S2B (left panel)

CUL4B

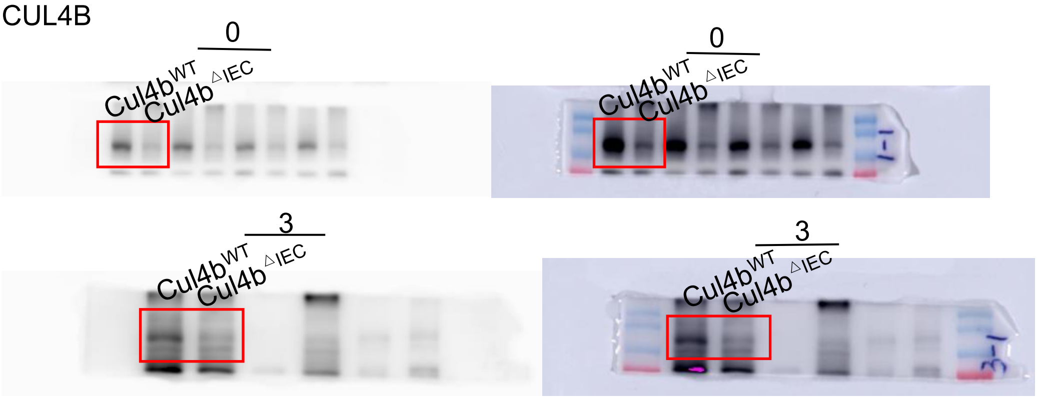


p-TBK1

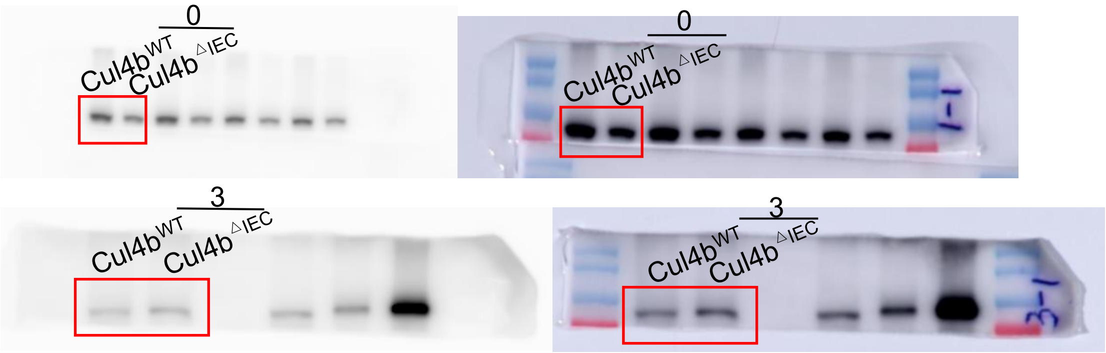


STING and p-STING

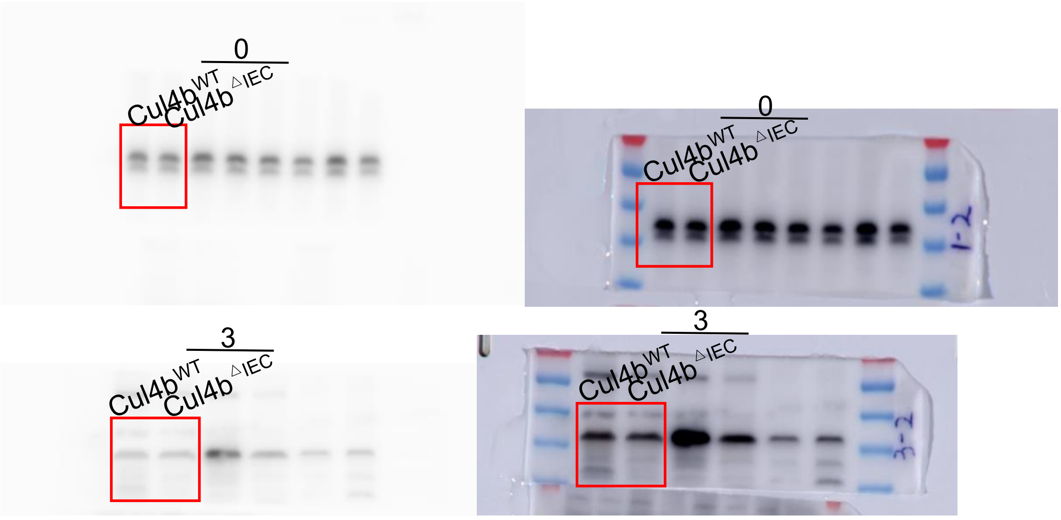


GAPDH

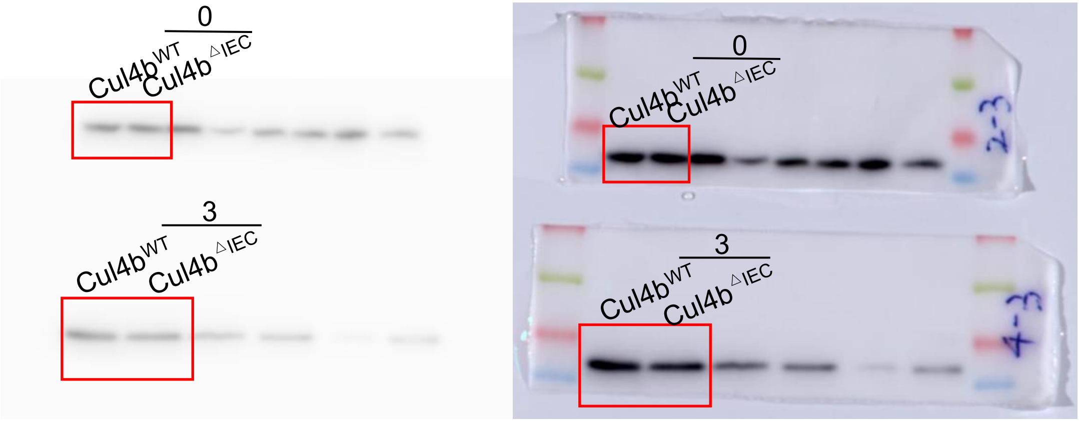
0
Cul4b$^{WT}$
Cul4b$^{\triangle IEC}$
3
Cul4b$^{WT}$
Cul4b$^{\triangle IEC}$
0
Cul4b$^{WT}$
Cul4b$^{\triangle IEC}$
2-3
3
Cul4b$^{WT}$
Cul4b$^{\triangle IEC}$
4-3

Figure S2 (right panel)

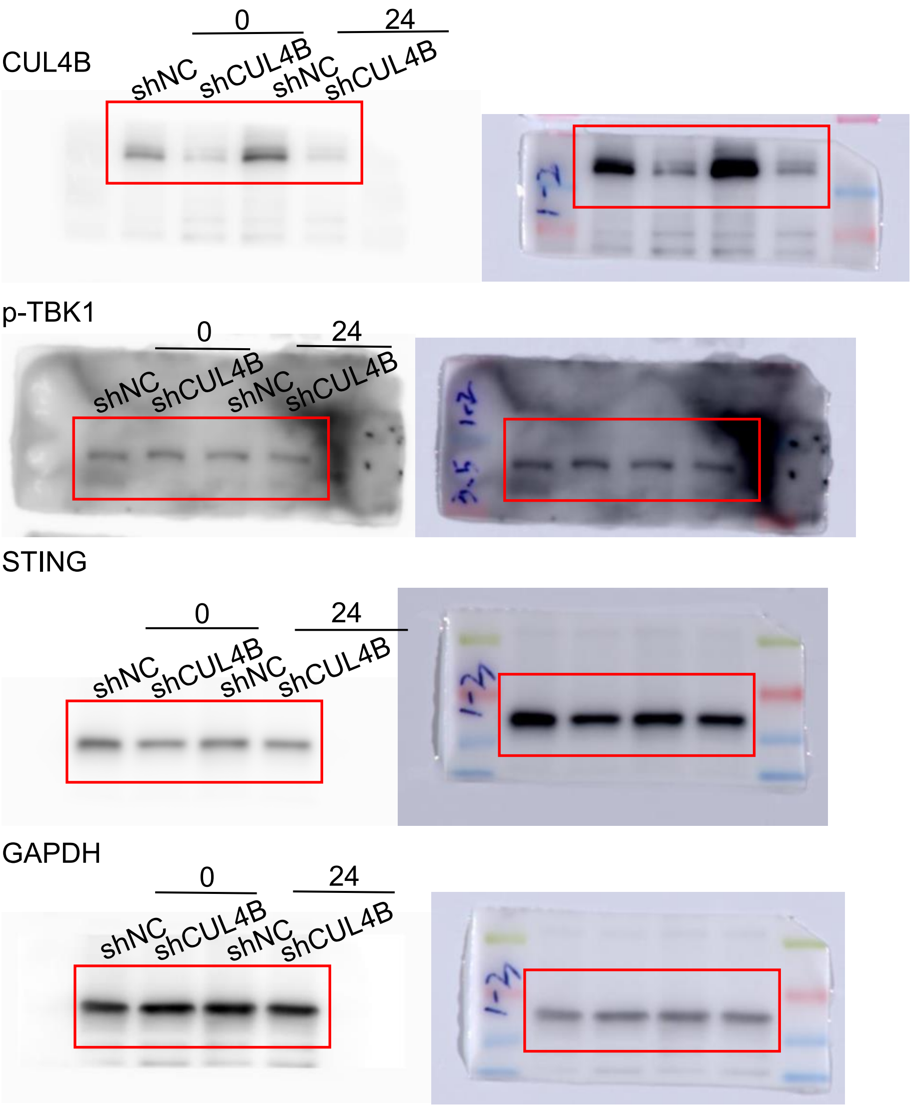

Figure S5A

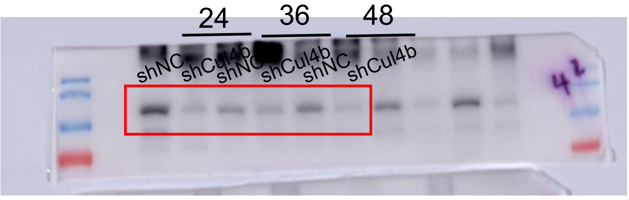


CUL4B

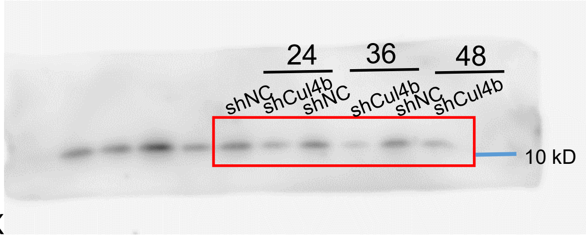


Gamma-H2AX

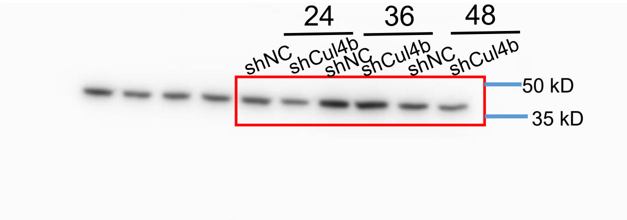


GAPDH

Figure S5C

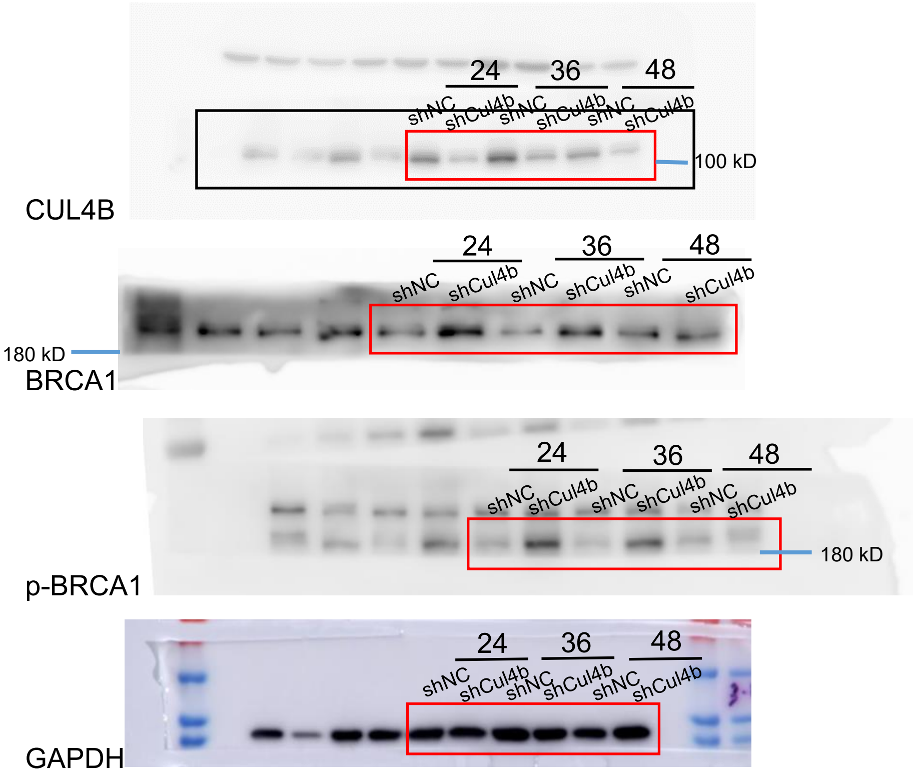

CUL4B
24
36
48
shNC
shCul4b
shNC
shCul4b
shNC
shCul4b
100 kD
BRCA1
180 kD
p-BRCA1
180 kD
GAPDH

Figure S5E

CUL4B

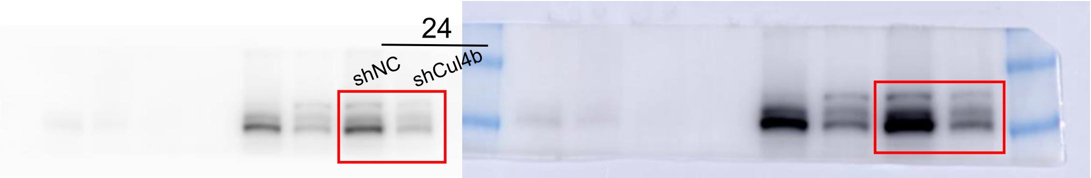


RAD51

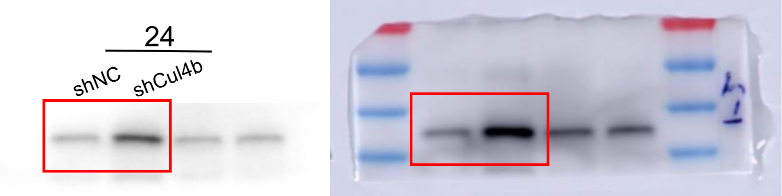


GAPDH

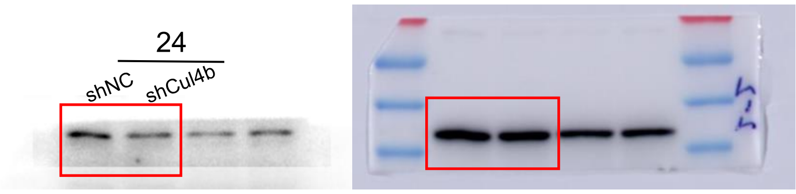


**Figure S6. Original gels of all images of Western blot results**

These are the original images with full-length membranes. The blots were cut prior to hybridisation with antibodies during blotting. Black line: the border of the blot; Red line: the samples used in manuscript.

Note: We used the “Incremental” mode to expose the membranes, resulting in the part of the original images without markers and clear edges. We restored the position of the markers according to the existing results using blue lines.
